# A Genome-Scale Atlas Reveals Complex Interplay of Transcription and Translation in an Archaeon

**DOI:** 10.1128/msystems.00816-22

**Published:** 2023-03-13

**Authors:** Alan P. R. Lorenzetti, Ulrike Kusebauch, Lívia S. Zaramela, Wei-Ju Wu, João P. P. de Almeida, Serdar Turkarslan, Adrián L. G. de Lomana, José V. Gomes-Filho, Ricardo Z. N. Vêncio, Robert L. Moritz, Tie Koide, Nitin S. Baliga

**Affiliations:** a Department of Biochemistry and Immunology, Ribeirão Preto Medical School, University of São Paulo, Ribeirão Preto, Brazil; b Institute for Systems Biology, Seattle, Washington, USA; c Department of Computation and Mathematics, Faculty of Philosophy, Sciences and Letters at Ribeirão Preto, University of São Paulo, Ribeirão Preto, Brazil; d Department of Biology, University of Washington, Seattle, Washington, USA; e Department of Microbiology, University of Washington, Seattle, Washington, USA; f Molecular and Cellular Biology Program, University of Washington, Seattle, Washington, USA; g Lawrence Berkeley National Lab, Berkeley, California, USA; Wageningen University

**Keywords:** *Archaea*, SmAP1, gas vesicles, gene expression, long-read DNA-Seq, mobile genetic elements, post-transcriptional RNA-binding proteins, post-transcriptional control mechanisms, proteomics, Web resource

## Abstract

The scale of post-transcriptional regulation and the implications of its interplay with other forms of regulation in environmental acclimation are underexplored for organisms of the domain *Archaea*. Here, we have investigated the scale of post-transcriptional regulation in the extremely halophilic archaeon Halobacterium salinarum NRC-1 by integrating the transcriptome-wide locations of transcript processing sites (TPSs) and SmAP1 binding, the genome-wide locations of antisense RNAs (asRNAs), and the consequences of RNase_2099C knockout on the differential expression of all genes. This integrated analysis has discovered that 54% of all protein-coding genes in the genome of this haloarchaeon are likely targeted by multiple mechanisms for putative post-transcriptional processing and regulation, with about 20% of genes likely being regulated by combinatorial schemes involving SmAP1, asRNAs, and RNase_2099C. Comparative analysis of mRNA levels (transcriptome sequencing [RNA-Seq]) and protein levels (sequential window acquisition of all theoretical fragment ion spectra mass spectrometry [SWATH-MS]) for 2,579 genes over four phases of batch culture growth in complex medium generated additional evidence for the conditional post-transcriptional regulation of 7% of all protein-coding genes. We demonstrate that post-transcriptional regulation may act to fine-tune specialized and rapid acclimation to stressful environments, e.g., as a switch to turn on gas vesicle biogenesis to promote vertical relocation under anoxic conditions and modulate the frequency of transposition by insertion sequence (IS) elements of the IS*200*/IS*605*, IS*4*, and IS*H3* families. Findings from this study are provided as an atlas in a public Web resource (https://halodata.systemsbiology.net).

**IMPORTANCE** While the transcriptional regulation landscape of archaea has been extensively investigated, we currently have limited knowledge about post-transcriptional regulation and its driving mechanisms in this domain of life. In this study, we collected and integrated omics data from multiple sources and technologies to infer post-transcriptionally regulated genes and the putative mechanisms modulating their expression at the protein level in Halobacterium salinarum NRC-1. The results suggest that post-transcriptional regulation may drive environmental acclimation by regulating hallmark biological processes. To foster discoveries by other research groups interested in the topic, we extended our integrated data to the public in the form of an interactive atlas (https://halodata.systemsbiology.net).

## INTRODUCTION

By virtue of their coexistence with multiple organisms within a community, microbes are under significant evolutionary selection pressure to maximize resource utilization for growth and sustenance while minimizing waste (1). For this reason, even within their streamlined genomes, microbes possess extensive regulatory mechanisms at multiple levels of information processing (2–5). While regulation at the transcriptional level is typically modular, with genome-wide consequences (4, 6), regulation at the post-transcriptional level is believed to be more nuanced and localized to specific sets of functions that are directly associated with environment-specific phenotypic traits (7). In other words, while transcriptional regulation mediates large-scale physiological adjustments, post-transcriptional regulation fine-tunes specific functions to optimize environmental acclimation. Understanding the interplay of regulation across the different layers of information processing will give insight into how microbes compete and collaborate effectively with other coinhabiting organisms. In addition to having foundational significance, these insights also have important implications for synthetic biology approaches to introduce novel traits while minimizing fitness trade-offs in an engineered organism (8–11).

Understanding the interplay of regulation across transcription and translation in organisms of the domain *Archaea* is especially interesting for several reasons. First, while they have been discovered across diverse environments, archaea are particularly known for specialized phenotypic adaptations to some of the most extreme and dynamic habitats (12). Second, archaea are unique in terms of possessing a mix of information-processing mechanisms that are distinctly eukaryotic or bacterial. For instance, while their general transcriptional machinery, including the RNA polymerase, shares ancestry with their eukaryotic counterparts, the regulation of transcription is mediated by regulators that have bacterial ancestry (13, 14). There has been extensive work across several archaeal model organisms that characterized basal transcription and its regulation both in molecular detail and at a systems level (2, 3, 15). In contrast, it has been only recently that we have begun to appreciate the role of post-transcriptional regulatory mechanisms in the specialized phenotypic acclimation of archaea. There is evidence that the translational efficiency (TE) in methanogenic archaea is modulated by the differential processing of 5′ untranslated regions (UTRs) (16), mRNA secondary structures (17), or context-specific binding by small regulatory RNAs (sRNAs) to conditionally occlude ribosome-binding sites within transcripts (18) or to stabilize them (19). A study conducted on a psychrophile discovered that post-transcriptional regulation directly influences methanol conversion into methane at lower temperatures (20). Similarly, in halophiles, RNase-mediated disruption of positive autoregulation of potassium uptake was discovered to be an important mechanism for energetically efficient and rapid acclimation in a salinity shift scenario (21). Moreover, the interaction between an sRNA and its target is crucial for resistance to oxidative stress (22). These examples illustrate how some archaea utilize post-transcriptional regulation to fine-tune specific functions and pathways for specialized phenotypic acclimation to environmental change.

However, much remains to be understood regarding the scale of post-transcriptional regulation in archaea and the extent to which it is deployed in combinatorial schemes to fine-tune phenotypes for environmental acclimation. For instance, the widely conserved and extensively characterized RNA-binding proteins (RBPs), including Csp (A, C, and E), CsrA, RNase E, YbeY, and Hfq, are known to play important post-transcriptional regulatory roles in bacteria (23), but there is a limited understanding of the roles of their orthologs in archaea. Hfq is a member of an RNA-guided complex, a well-characterized bacterial RNA chaperone known to interfere with mRNA translation (24, 25), which acts in a manner analogous to that of the RNA-induced silencing complex (RISC) in eukaryotes to regulate specific mRNAs (26). Notably, the Hfq homolog, Sm-like archaeal protein 1 (SmAP1) (or Lsm), has been characterized structurally across multiple archaea (27–30), including Halobacterium salinarum NRC-1 (31), and was shown to likely mediate post-transcriptional regulation through sRNA binding in Haloferax volcanii (32, 33) and Sulfolobus solfataricus (34). Yet we do not fully understand the mechanism, importance, context, or scale of post-transcriptional regulation mediated by SmAP1 (and other RBPs) (35, 36) or, for that matter, by the large numbers of sRNAs, antisense RNAs (asRNAs), and RNases that have been discovered across archaeal genomes (37).

Here, we have investigated the scale of the interplay between transcriptional and post-transcriptional mechanisms in regulating protein levels in the halophilic archaeon H. salinarum NRC-1, which has served as a model to investigate the traits of organisms in the domain *Archaea*. In particular, *H. salinarum* NRC-1 has been widely used as a model organism to dissect hallmark traits of halophilic archaea, including niche adaptation via expanded families of general transcription factors (38), large-scale genome organization by genomic repeats and insertion sequences (ISs) (39, 40), flotation by gas vesicle biogenesis (41), phototransduction by bacteriorhodopsin (42), and how the modularity of translational complexes enables rapid acclimation to environmental changes (43). Previous work characterized many aspects of the global transcriptional regulatory network of *H. salinarum* NRC-1 at a systems level and in mechanistic detail (2, 3), with extensive validations through genetic perturbation studies and physical mapping of genome-wide protein-DNA interactions of multiple transcription factors (4, 5). However, the transcriptional regulatory network by itself or the half-lives of all transcripts (44) did not fully explain the complex relationship between the absolute and relative abundances of transcripts and proteins across different environmental contexts (45, 46), suggesting an important role for post-transcriptional regulation. Indeed, previous studies have uncovered evidence of the potential for extensive post-transcriptional regulation in *H. salinarum* NRC-1, including the presence of a strikingly large number of regulatory elements within coding sequences (3), which leads to the widespread conditional splitting of at least 40% of all operons into multiple overlapping transcriptional units (5), the presence of asRNAs for 22% of all genes (47), the differential regulation of 23 transcripts in an RNase knockout background (21), and extensive transcript processing sites (TPSs) across 43% of all coding sequences (48).

Through an integrated analysis of a new transcriptome-wide map of SmAP1 binding located by RNA immunoprecipitation sequencing (RIP-Seq), the differential expression reanalysis of a transcriptome data set generated upon the deletion of an RNase (VNG_2099C) implicated in acclimation to salinity changes (21), and the location of previously mapped asRNAs and TPSs (47, 48), we have generated a genome-scale atlas that has led to the discovery that 54% of all protein-coding genes in *H. salinarum* NRC-1 are targeted by multiple mechanisms for putative post-transcriptional regulation. Interestingly, 20% of all protein-coding genes are likely post-transcriptionally regulated in combinatorial schemes involving SmAP1, asRNAs, and RNase. Furthermore, through a comparative reanalysis of publicly available data sets, we investigated dynamic changes in mRNA levels (transcriptome sequencing [RNA-Seq]), ribosome footprints (ribosome sequencing [Ribo-Seq]) (43), and protein levels (sequential window acquisition of all theoretical fragment ion spectra mass spectrometry [SWATH-MS]) (U. Kusebauch et al., unpublished data) for 2,579 representative genes over four phases of batch culture growth in complex medium (CM). We generated evidence that 7% of all protein-coding genes (188 genes) are indeed post-transcriptionally regulated. Notably, 78% of these post-transcriptionally regulated genes were mechanistically associated with SmAP1 binding, asRNA, TPS, and/or RNase-mediated differential regulation. Through an in-depth analysis, we demonstrate how post-transcriptional regulation acts to fine-tune specialized environmental acclimation, e.g., as a switch to turn on gas vesicle biogenesis and modulate the frequency of transposition by IS elements of the IS*200*/IS*605*, IS*4*, and IS*H3* families. Finally, we have generated an interactive Web resource to support the collaborative community-wide exploration and characterization of the *H. salinarum* NRC-1 multi-omics atlas (https://halodata.systemsbiology.net).

## RESULTS

### Evidence for post-transcriptional regulation by SmAP1, asRNAs, and RNase_2099C.

Since the publication of its genome sequence in 2000, multiple sources of gene annotations have emerged for *H. salinarum* NRC-1 (49–51). To standardize annotations, we clustered sequences from each source to eliminate redundancy while differentiating between paralogs (see Materials and Methods; see also Data Set S1 in the supplemental material and Table S1 at https://doi.org/10.6084/m9.figshare.21936396.v2). In summary, this analysis identified 2,631 nonredundant transcripts, including 2,579 coding and 52 noncoding RNAs (rRNAs, tRNAs, signal recognition particle RNA, and RNase P), with a dictionary anchored by locus tags described previously (51) and mapped to locus tags of the closely related strain *H. salinarum* R1 (Data Set S1).

10.1128/msystems.00816-22.9 DATA SET S1 Atlas data. The nonredundant transcriptome locus tag dictionary, the normalized atlas data, and the nonnormalized atlas data are included. Download Data Set S1, XLSX file, 3.0 MB. Copyright © 2023 Lorenzetti et al. 2023 Lorenzetti et al. https://creativecommons.org/licenses/by/4.0/ This content is distributed under the terms of the Creative Commons Attribution 4.0 International license.

Next, we compiled orthogonal, genome-wide evidence for putative post-transcriptional regulation. Specifically, we (i) assigned published predicted transcript processing sites (TPSs), acquired through a search for the enrichment of monophosphorylated RNAs (nonprimary transcripts) in a differential RNA-Seq (dRNA-Seq) experiment (48), to at least 966 protein-coding genes (37% of all protein-coding genes); (ii) mapped previously annotated *cis*-acting asRNAs for 536 genes (47); and (iii) determined, from the sole publicly available RNase knockout transcriptome data set, that 166 genes were differentially expressed upon the deletion of 1 out of 13 RNases predicted within the genome (VNG_2099C [“RNase_2099C” here]) (21) (see Data Set S2 at https://doi.org/10.6084/m9.figshare.21936399.v2). To characterize the role of SmAP1 (VNG_1496G) in *H. salinarum* NRC-1, epitope-tagged SmAP1-RNA complexes were coimmunoprecipitated from late-exponential-phase cultures under standard growth conditions (Fig. S1A and B), and the transcriptome-wide binding locations of SmAP1 were mapped by the enrichment of sequenced transcripts (RIP-Seq) (see Materials and Methods). Consistent with previous *in vitro* observations of diverse archaea, the RIP-Seq analysis led to the discovery that SmAP1 preferentially binds to AU-rich transcripts (Fig. S1C) (29–32, 52). In particular, we determined that SmAP1 binds to 15% (397/2,579) of all protein-coding transcripts in *H. salinarum* NRC-1, including its own coding transcript (Data Set S1), suggesting putative autoregulation in light of the observed dynamics for mRNA and protein levels (Fig. S1D).

10.1128/msystems.00816-22.1 FIG S1 Quality assurance for coimmunoprecipitated samples. (A) Western blotting of samples extracted from strains expressing plasmids for cMyc and cMyc-tagged SmAP1 (see the lane titles for labels). The expected molecular weight of the cMyc-tagged SmAP1 complex is 37 kDa. BR, biological replicate. (B) PCR of RNA-purified samples treated with DNase. Lanes: M, ladder; 1, positive control (genomic DNA amplified using primers 19-fwd and 20-rev, with a predicted amplicon size of 85 bp); 2 to 5, cMyc BR1, cMyc BR2, SmAP1-cMyc BR1, and SmAP1-cMyc BR2, respectively (amplified using primers 19-fwd and 20-rev); 6, positive control (genomic DNA amplified using primers 63-fwd and 64-rev, with a predicted amplicon size of 450 bp); 7 to 10, cMyc BR1, cMyc BR2, SmAP1-cMyc BR1, and SmAP1-cMyc BR2, respectively (amplified using primers 63-fwd and 64-rev). (C to E) SmAP1 features. (C) SmAP1 binding is conditioned to the GC content of transcripts. The reduced GC content of transcripts is a property that influences SmAP1 binding. We compared medians using the Mann-Whitney U test. ****, *P* ≤ 10^−4^. (D) Time course view of protein, ribosome-protected mRNA fragment (RPF) (TPM+1), and mRNA levels (TPM+1). Vertical bars represent the standard errors computed using at least six replicates for proteins and three replicates for mRNAs and RPFs. (E) Functional categories of transcripts bound to SmAP1. Shown are the numbers of genes that have transcripts bound to SmAP1, considering each COG (clusters of orthologous genes) category. The left-hand side shows categories with no more than 25 genes with SmAP1-bound transcripts, and the right-hand side shows genes within the “Function unknown” category. We highlight enriched categories with an asterisk (*, *P* < 0.05). Download FIG S1, TIF file, 2.3 MB. Copyright © 2023 Lorenzetti et al. 2023 Lorenzetti et al. https://creativecommons.org/licenses/by/4.0/ This content is distributed under the terms of the Creative Commons Attribution 4.0 International license.

**FIG 1:**
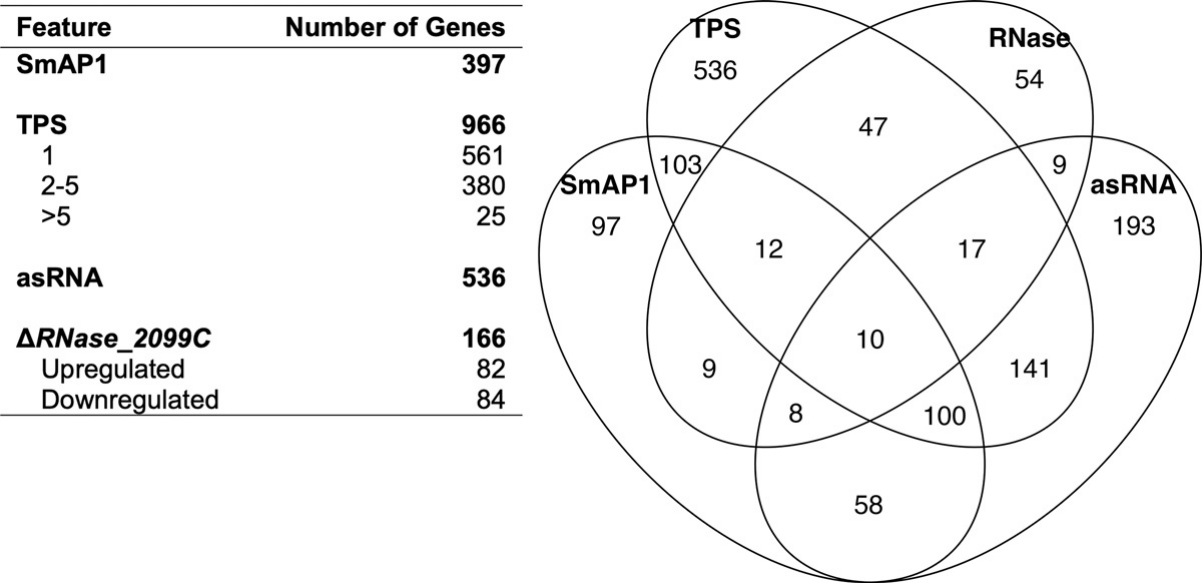
Features potentially associated with post-transcriptional regulation. Four features related to post-transcriptional regulation in *H. salinarum* are shown. Sets are comprised of genes that bind to SmAP1, show transcript processing sites (TPSs), have a putative *cis*-regulatory antisense RNA (asRNA), and are differentially expressed in the RNase_2099C knockout strain (Δ*RNase_2099C*).

An integrated analysis of the locations of SmAP1 binding, asRNAs, and TPSs and differential expression in the Δ*RNase_2099C* strain revealed that at least 1,394 genes were potentially subject to post-transcriptional regulation by at least one of these mechanisms, with 514 genes being under putative combinatorial regulation by two or more mechanisms (Fig. 1). Interestingly, transcripts that were upregulated in the Δ*RNase_2099C* strain background were preferentially bound by SmAP1 (*P* = 0.02), associated with cognate asRNAs (*P* = 0.04), and enriched for TPSs (*P* = 6.7 × 10^−5^). These findings suggest that SmAP1 and asRNAs are responsible for the recruitment of RNase_2099C to mediate the targeted cleavage of transcripts. Thus, the integrated analysis predicted that 20% to 54% of the *H. salinarum* genome is post-transcriptionally regulated (514 to 1,394 out of 2,579 genes) (Fig. 1). The fact that SmAP1, asRNAs, and RNase_2099C account for putative regulation of 858 genes suggests that myriad mechanisms, potentially involving other RBPs and RNases noted above, are likely at play, even under the limited conditions represented by standard growth conditions.

### Evidence of post-transcriptional regulation in global trends of mRNA and protein levels.

**FIG 2:**
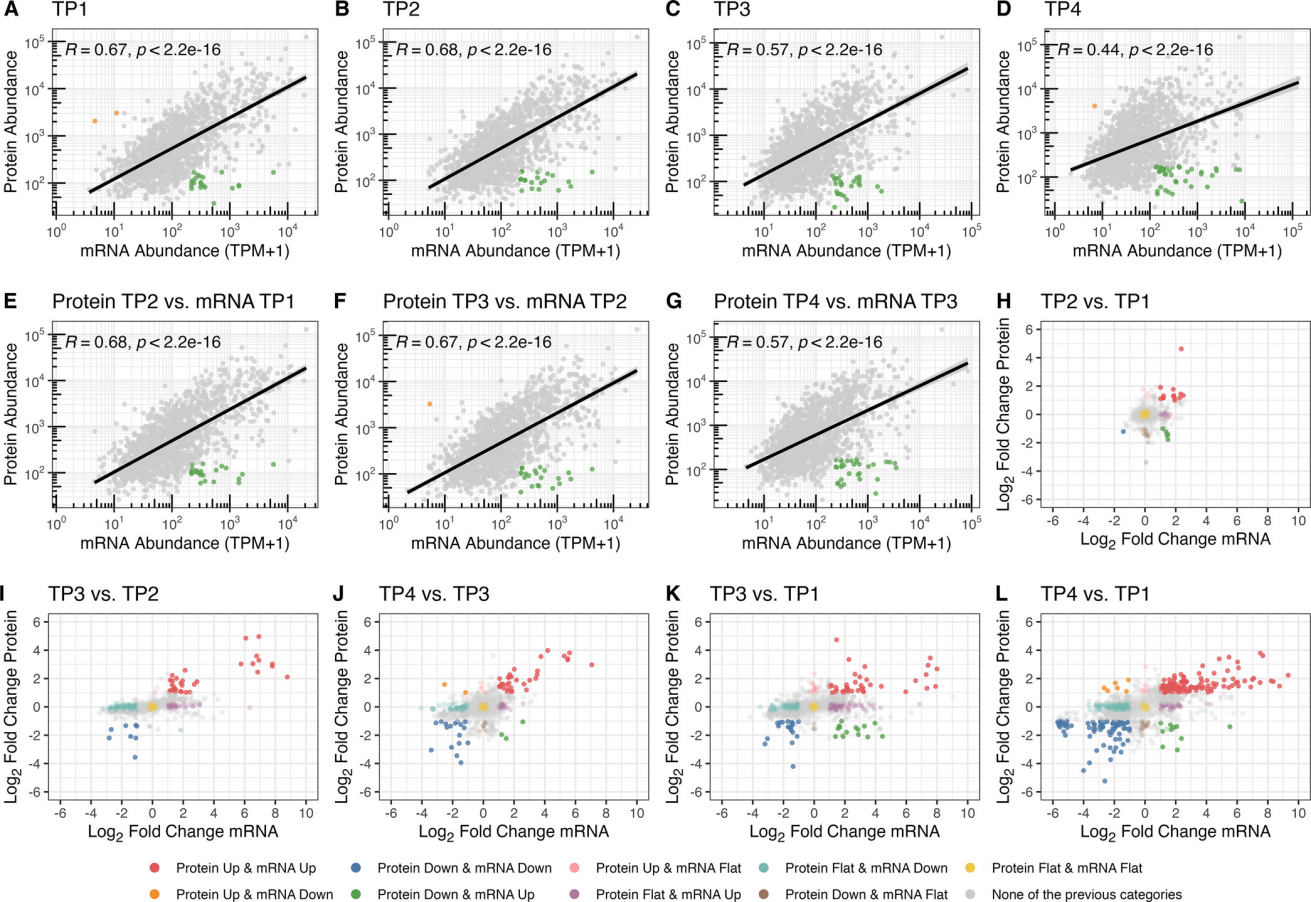
Genes following patterns compatible with post-transcriptional regulation. Each panel shows protein (*y* axis) and mRNA (*x* axis) absolute abundances (log_10_ transformed) or relative changes (log_2_ fold changes). (A to G) Absolute-abundance-based analysis in a time-point-wise manner (A to D) and from a time lag perspective (E to G). Gray points represent entities following the usual patterns, orange points represent entities within the upper quintile of protein abundance and the lower quintile of mRNA abundance, and green points represent entities within the lower quintile of protein abundance and the upper quintile of mRNA abundance. The solid black line illustrates the fitted linear regression model. (H to L) Relative-abundance-based analysis of protein and mRNA levels in consecutive physiological state transitions (H to J) and the same variables for long physiological state transitions (K and L). Points are color-coded according to multiple combinations of status change considering both variables. TP1, early exponential growth phase; TP2, mid-exponential growth phase; TP3, late exponential growth phase; TP4, stationary phase.

We reanalyzed a previously published transcriptome data set (43) obtained by RNA-Seq and integrated it with proteome data (Kusebauch et al., unpublished) obtained for the same samples by sequential window acquisition of all theoretical fragment ion spectra (SWATH), a data-independent acquisition method combining comprehensive identification with reproducible quantitation of peptides and proteins by mass spectrometry (MS). We investigated the concordance in the patterns of absolute abundances at the transcriptional and translational levels for each gene by calculating Pearson correlation coefficients between mRNA and protein quantities across all of the sampled physiological states (Pearson correlation coefficient for the early exponential phase [time point 1] [*R*_TP1_] = 0.67; Pearson correlation coefficient for the mid-exponential phase [*R*_TP2_] = 0.68; Pearson correlation coefficient for the late exponential phase [*R*_TP3_] = 0.57; Pearson correlation coefficient for the stationary phase [*R*_TP4_] = 0.44) (Fig. 2A to D). The weaker correlation (*R*_TP1_ = *R*_TP2_ > *R*_TP3_ > *R*_TP4_) (see Table S2 at https://doi.org/10.6084/m9.figshare.21936396.v2) in the later stages of batch culture growth was skewed toward the repression of translation; that is, highly abundant mRNAs were associated with low-abundance proteins in the quiescent physiological state (TP4). We also noticed that protein levels correlated slightly better with mRNA levels from the previous time point (*R*_P-TP2 m-TP1_ = 0.68; *R*_P-TP3 m-TP2_ = 0.67; *R*_P-TP4 m-TP3_ = 0.57) (Fig. 2E to G; see also Table S2 at https://doi.org/10.6084/m9.figshare.21936396.v2), which is consistent with the sequential and temporal relationship between transcription and translation, as we have previously shown (45, 46). We discovered that 6.5% of all protein-coding genes (167) with high mRNA levels (upper quintile) were associated with low protein levels (lower quintile or undetected) over some or all four stages of growth in batch culture (Fig. S2A; see also Data Set S3 at https://doi.org/10.6084/m9.figshare.21936399.v2). Specifically, the 167 genes were enriched for SmAP1 binding, asRNAs, and TPSs (*P* = 2.3 × 10^−4^, 2.9 × 10^−2^, and 1.1 × 10^−7^, respectively) and had longer average mRNA half-lives (13.7 min versus 12.3 min; *P* = 1.1 × 10^−2^). Within this set, 64 genes associated with protein levels detected in the lower quintile (Fig. 2A to D, green points, and Fig. S2B; see also Data Set S3 at https://doi.org/10.6084/m9.figshare.21936399.v2) were enriched for TPSs (*P* = 2.6 × 10^−4^). A second set of 117 genes, whose proteins were not detected, likely due to their low levels or complete absence (see Materials and Methods) (Fig. S2C; see also Data Set S3 at https://doi.org/10.6084/m9.figshare.21936399.v2), was enriched for SmAP1 binding and TPSs (*P* = 1.7 × 10^−6^ and 2.8 × 10^−6^, respectively), had longer average mRNA half-lives (14.2 min versus 12.3 min; *P* = 2.7 × 10^−3^), and was upregulated in the Δ*RNase_2099C* strain (*P* = 1.5 × 10^−2^). See Data Set S4 at https://doi.org/10.6084/m9.figshare.21936399.v2 for sets and tests.

10.1128/msystems.00816-22.2 FIG S2 Venn diagrams of putative post-transcriptionally regulated genes shared among different physiological states. (A) Entities with proteins within the lower quintile of protein levels or not detected by our proteome survey whose mRNA levels are within the upper quintile (union set = 167). (B) Entities within the lower quintile of protein levels and the upper quintile of mRNA levels (union set = 64). (C) Entities with proteins not detected by our proteome survey and within the upper quintile of mRNA levels (union set = 117). TP1, early exponential growth phase; TP2, mid-exponential growth phase; TP3, late exponential growth phase; TP4, stationary phase. All sets are available in Data Set S3 at https://doi.org/10.6084/m9.figshare.21936399.v2. Download FIG S2, TIF file, 1.4 MB. Copyright © 2023 Lorenzetti et al. 2023 Lorenzetti et al. https://creativecommons.org/licenses/by/4.0/ This content is distributed under the terms of the Creative Commons Attribution 4.0 International license.

Finally, we searched for potentially post-transcriptionally regulated genes by correlating dynamic relative changes in protein and mRNA levels over time (Fig. 2H to L; see also Data Sets S5 and S6 at https://doi.org/10.6084/m9.figshare.21936399.v2). For example, during the transition from TP1 to TP2, we observed decreases in the protein abundances of five transcriptionally upregulated genes over the same time frame (Fig. 2H). This cluster (Fig. S3A; see also Data Set S6 at https://doi.org/10.6084/m9.figshare.21936399.v2), comprised of five genes (VNG_7025, VNG_7026, VNG_7039, VNG_7103, and VNG_6313G) (Fig. 2H, green points) with enrichment for SmAP1 binding, asRNAs, and TPSs (*P* = 8.5 × 10^−5^, 3.8 × 10^−4^, and 0, respectively), is a strong candidate for post-transcriptional repression. The genes also had a low codon adaptation index (CAI) (0.64 versus 0.77; *P* = 3.9 × 10^−3^) and increased mRNA levels in the Δ*RNase_2099C* strain (log_2_ fold change [LFC] of 1 versus 0.02; *P* = 3.5 × 10^−4^). The comparative analysis of the changes in mRNA and protein abundances across all transition states (TP1 to TP2, TP2 to TP3, TP3 to TP4, TP1 to TP3, and TP1 to TP4) identified 26 potentially post-transcriptionally repressed transcripts (Fig. S3B; see also Data Set S6 at https://doi.org/10.6084/m9.figshare.21936399.v2) enriched for SmAP1 binding and TPSs (*P* = 3.5 × 10^−3^ and 2.3 × 10^−4^, respectively) and upregulated in the Δ*RNase_2099C* strain (*P* = 9.2 × 10^−7^). Again, see Data Set S4 at https://doi.org/10.6084/m9.figshare.21936399.v2 for sets and tests.

10.1128/msystems.00816-22.3 FIG S3 (A) Atlas section of putative post-transcriptionally regulated genes in the transition from TP1 to TP2. This section of the atlas shows genes having downregulated proteins and upregulated mRNAs (green cluster in Fig. 2H) in the transition from the early exponential growth phase (TP1) to the mid-exponential growth phase (TP2). The heat map represents the log_10_-transformed expression profile of proteins (a pseudocount was imputed for missing values), mRNAs (TPM+1), and ribosome-protected mRNA fragments (RPFs) (TPM+1). Heat maps also represent the respective log_2_-transformed translational efficiency (TE) and ribosome occupancy (RO) values for each time point. COG, clusters of orthologous genes; asRNAs, antisense RNAs; TPS, transcript processing site; 2099, log_2_ fold change (LFC) of transcripts in the absence of RNase_2099C; CAI, codon adaptation index; TP3, late exponential growth phase; TP4, stationary phase. (B) UpSet plot of putative post-transcriptionally regulated genes shared in different physiological state transitions. Entities are downregulated at the protein level and upregulated at the mRNA level (union set = 26). All sets are available in Data Set S6 at https://doi.org/10.6084/m9.figshare.21936399.v2. Download FIG S3, TIF file, 0.9 MB. Copyright © 2023 Lorenzetti et al. 2023 Lorenzetti et al. https://creativecommons.org/licenses/by/4.0/ This content is distributed under the terms of the Creative Commons Attribution 4.0 International license.

Altogether, the combined analyses of correlations between the absolute and relative abundances of mRNAs and proteins provided further evidence for the post-transcriptional regulation of at least 7% of all genes (188 out of 2,579) in *H. salinarum* NRC-1 during the transition from active growth to the stationary phase. Notably, 78% of these genes (147/188) with poor mRNA-protein correlations were among the 1,394 genes associated with putative post-transcriptional regulation features, including SmAP1 binding, asRNAs, and TPSs (*P* = 1.9 × 10^−9^, 7.6 × 10^−6^, and 2.5 × 10^−21^, respectively). Together, these findings suggest the complex combinatorial post-transcriptional regulation of these genes at specific growth stages.

### Construction of the *H. salinarum* NRC-1 multi-omics atlas.

**FIG 3:**
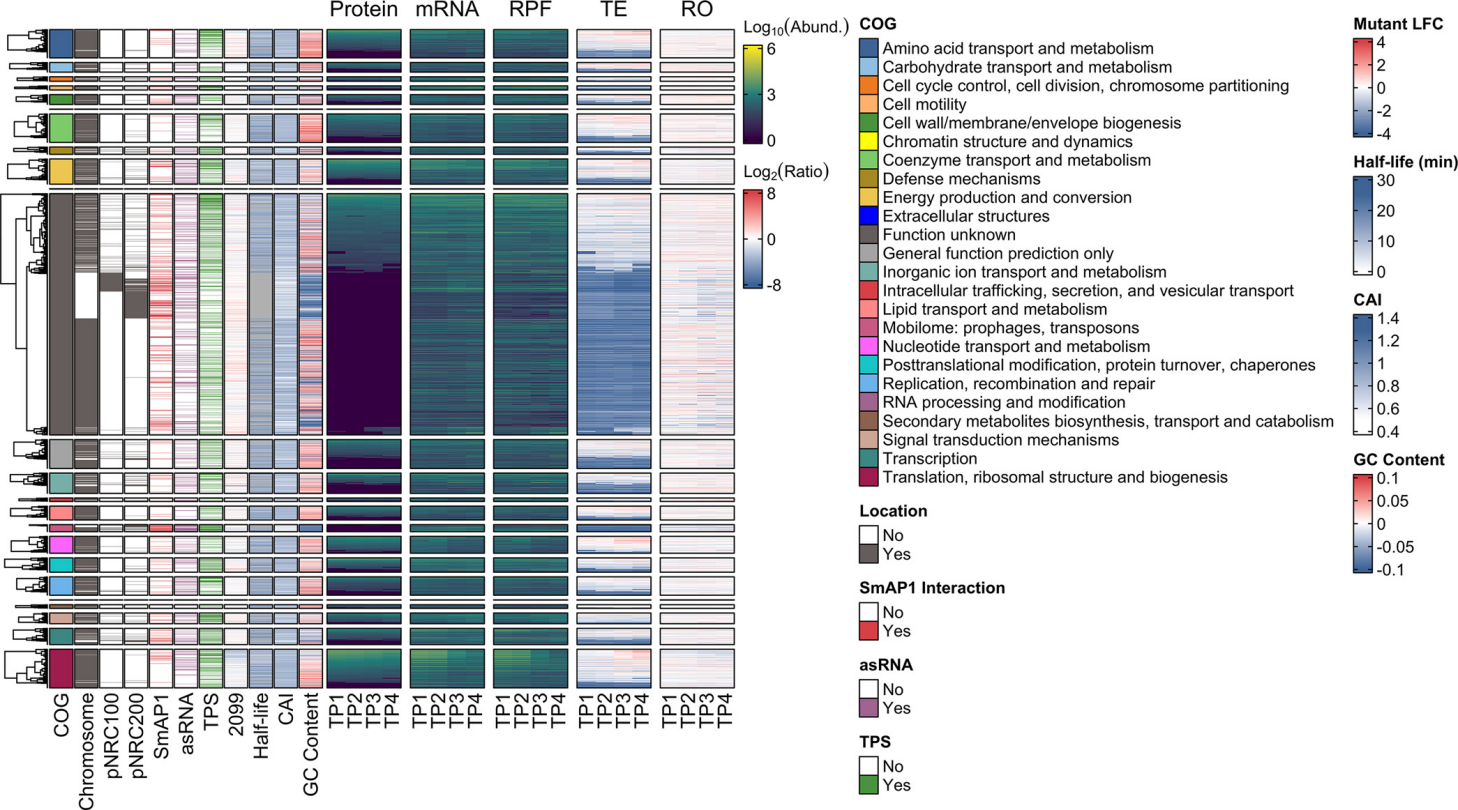
An atlas of the transcriptome, ribosome profile, and proteome for Halobacterium salinarum NRC-1. The heat map shows quantile-normalized log_10_-transformed abundances of proteins (a pseudocount was imputed for missing values), mRNAs (TPM+1), and ribosome-protected mRNA fragments (RPFs) (TPM+1) for 2,579 genes across four consecutive stages of batch culture growth, namely, early exponential, mid-exponential, late exponential, and stationary phases (TP1, TP2, TP3, and TP4, respectively). Log_2_-transformed translational efficiency (TE) and ribosome occupancy (RO) values were computed by dividing protein levels by mRNA levels and mRNA levels by RPF levels, respectively. We present general features on the left-hand side, starting with the clusters of orthologous genes (COG) functional categories (98), split into groups before clustering the protein levels. Chromosome, pNRC100, and pNRC200 show the replicon location of each gene within the genome. The presence of SmAP1 binding, antisense RNAs (asRNAs) (47), and putative endoribonuclease-generated transcript processing sites (TPSs) (48) is indicated in the corresponding tracks. The 2099 track shows the log_2_ fold changes (LFCs) in transcript levels in the RNase_2099C-null mutant (Δ*RNase_2099C*) relative to those in the parent Δ*ura3* strain (21). mRNA half-lives (44), the codon adaptation indices (CAIs), and the deviation of the GC content from the average GC content of all transcripts are also indicated in the corresponding tracks. See the keys for color codes for each track, and see Materials and Methods for details. Interactive and expanded static versions of this figure are available in our *H. salinarum* NRC-1 multi-omics atlas portal (https://halodata.systemsbiology.net).

To facilitate the discovery of evidence of post-transcriptional regulation, we compiled the corresponding quantities of mRNAs (RNA-Seq), ribosome-protected mRNA fragments (RPFs) (Ribo-Seq) (43), and proteins (SWATH-MS) (Kusebauch et al., unpublished) for 2,579 genes across the early exponential (TP1), mid-exponential (TP2), late exponential (TP3), and stationary (TP4) phases of growth in batch culture (see Materials and Methods) (Fig. 3; see also Data Set S7 at https://doi.org/10.6084/m9.figshare.21936399.v2). We obtained average values from replicates to represent each physiological state. Next, for each time point, we quantile normalized (Data Set S1) each data set for scale adjustment. Subsequently, we calculated the translational efficiency (TE) by dividing protein levels by mRNA levels, and we calculated ribosome occupancy (RO) by dividing the numbers of RPFs by mRNA levels. Finally, along with the SmAP1-binding status, the presence of asRNAs and TPSs, and the differential regulation of RNase_2099C, we included general properties such as the GC (guanine-cytosine) content, mRNA half-life, and CAI for each gene. These features are known to influence the dynamics of the interplay between transcription and translation (44, 53) and could explain the discrepant patterns of the corresponding changes across mRNAs, RPFs, and proteins. Genes in the atlas were organized into nine groups based on patterns of absolute abundance (see Data Set S3 at https://doi.org/10.6084/m9.figshare.21936399.v2) and relative changes across mRNA and protein levels (see Data Set S6 at https://doi.org/10.6084/m9.figshare.21936399.v2). The *H. salinarum* NRC-1 atlas is accessible through an application (https://halodata.systemsbiology.net) that supports interactive exploration by zooming in on specific segments of a heat map, by searching for genes of interest, or by using a searchable genome browser. The following sections demonstrate how the atlas facilitates in-depth investigations into the post-transcriptional regulation of hallmark processes in *H. salinarum* NRC-1.

### Functional implications of growth-associated post-transcriptional regulation in *H. salinarum*.

**FIG 4:**
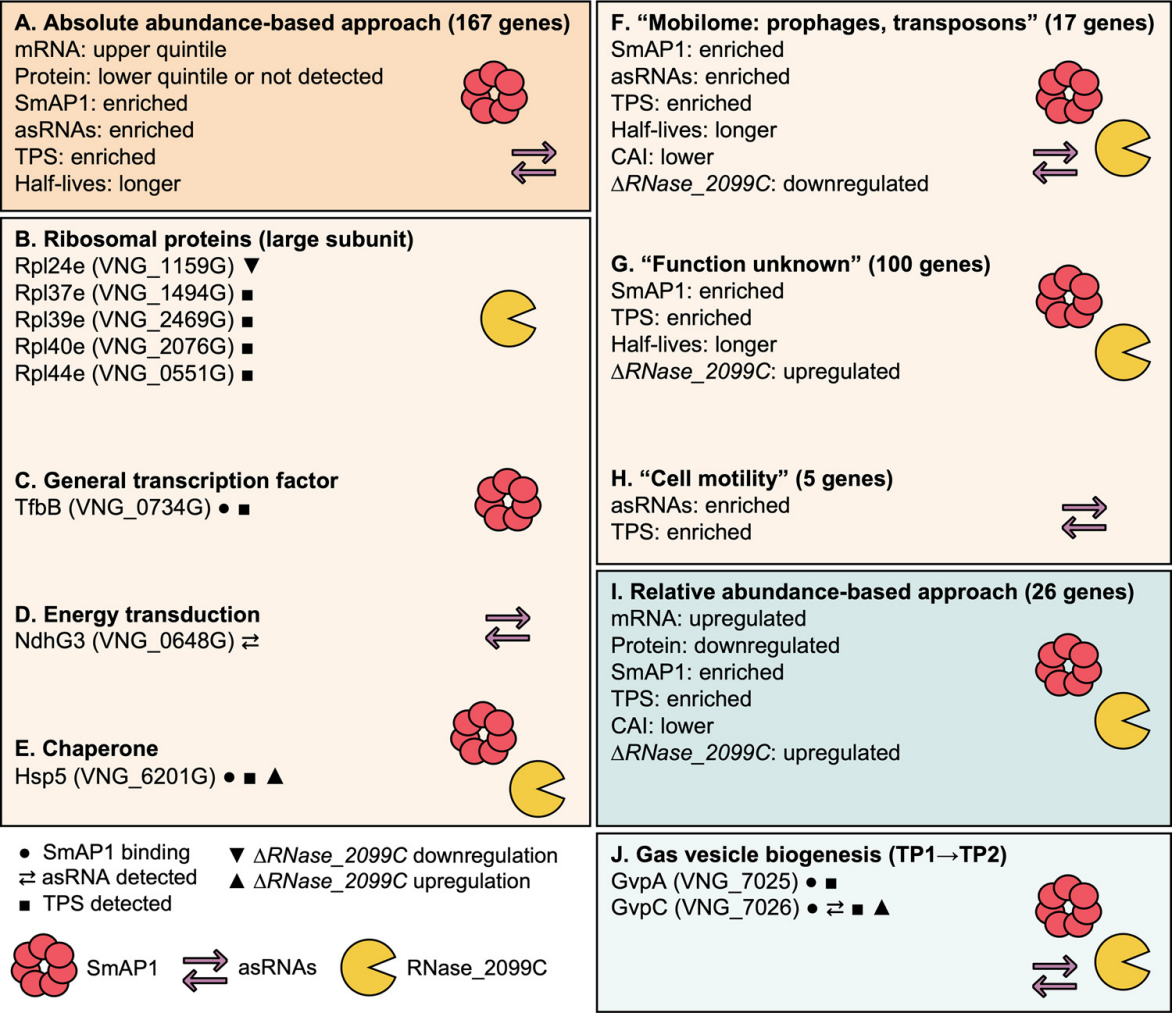
Functions of putative post-transcriptionally regulated genes and potential driving mechanisms. The common properties of groups of putative post-transcriptionally regulated genes are shown. (A) The union set of genes found by the absolute-abundance-based approach across the growth curve (green points in Fig. 2A to D). (B to E) Arbitrarily selected genes of known functions (subsets of those in panel A). (F to H) Gene categories according to clusters of orthologous genes (COGs) with enriched features compatible with the post-transcriptional regulation hypothesis (subsets of the genes in panel A). (I) The union set of genes found by the relative-abundance-based approach across the growth curve (upregulated mRNA and downregulated protein) (green clusters in Fig. 2H to L). (J) Genes of the *gvp* cluster in the transition from the early exponential (TP1) to the mid-exponential (TP2) growth phase (subset of the genes in panel I). See Data Set S4 at https://doi.org/10.6084/m9.figshare.21936399.v2 for a complete list of genes within each group (A and F to I) and the respective supporting evidence. TPS, transcript processing site; asRNA, antisense RNA; CAI, codon adaptation index.

Altogether, the comparison of the absolute and relative abundances of mRNAs and proteins yielded evidence for the post-transcriptional regulation of 188 genes during batch culture growth (Fig. 2; see also Data Sets S3 and S6 at https://doi.org/10.6084/m9.figshare.21936399.v2). Furthermore, the longer transcript half-lives together with the enrichment of SmAP1 binding, asRNAs, and TPSs and differential regulation upon the deletion of RNase_2099C provided evidence for post-transcriptional processing and associated putative mechanisms of regulation in different gene subsets. While a substantial number of genes were of unknown function, important processes were represented among genes of known functions; these included gas vesicle biogenesis, transposition-mediated genome reorganization, motility, translation, and energy transduction (Fig. 4). Among these, both gas vesicles and extensive genome reorganization mediated by the activity of mobile genetic elements are hallmark traits of *H. salinarum* NRC-1 that are triggered in specific environmental contexts, including late growth and stationary phases. Below, we present vignettes on each of these two processes to illustrate how the *H. salinarum* NRC-1 multi-omics atlas enables the discovery of mechanistic insight into the post-transcriptional regulation of specific phenotypes.

### (i) Role of SmAP1 in the regulation of transposition and genome reorganization.

**FIG 5:**
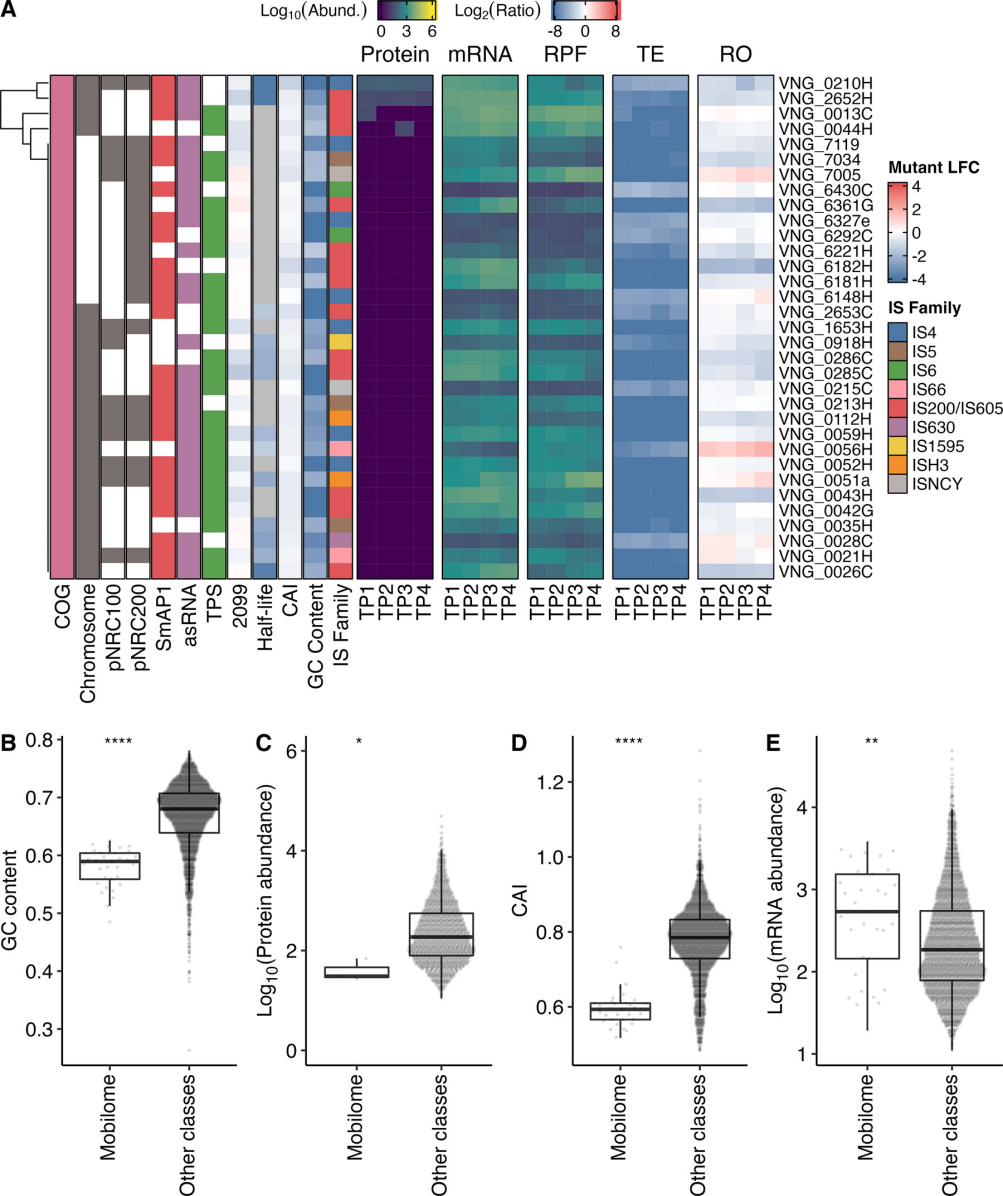
Protein and mRNA levels of mobile genetic elements. (A) Log_10_-transformed expression profile of proteins (a pseudocount was imputed for missing values), mRNAs (TPM+1), and ribosome-protected mRNA fragments (RPFs) (TPM+1) with miscellaneous properties of genes classified by clusters of orthologous genes (COG) within the “Mobilome: prophages, transposons” category (pink). TE, translational efficiency; RO, ribosome occupancy; asRNA, antisense RNA; TPS, transcript processing site; 2099, log_2_ fold change (LFC) of transcripts in the absence of RNase_2099C; TP1, early exponential growth phase; TP2, mid-exponential growth phase; TP3, late exponential growth phase; TP4, stationary phase. (B to E) Box plots aiding in the comparison of the features of genes within the “Mobilome: prophages, transposons” category versus the pool of the other categories. (B) GC content. (C) Log_10_-transformed average protein abundances across all time points (missing values are excluded). (D) Codon adaptation index (CAI). (E) Log_10_-transformed average mRNA levels (TPM+1) across all time points. We compared medians using the Mann-Whitney U test. *, *P* ≤ 5 × 10^−2^; **, *P* ≤ 10^−2^; ****, *P* ≤ 10^−4^.

**FIG 6:**
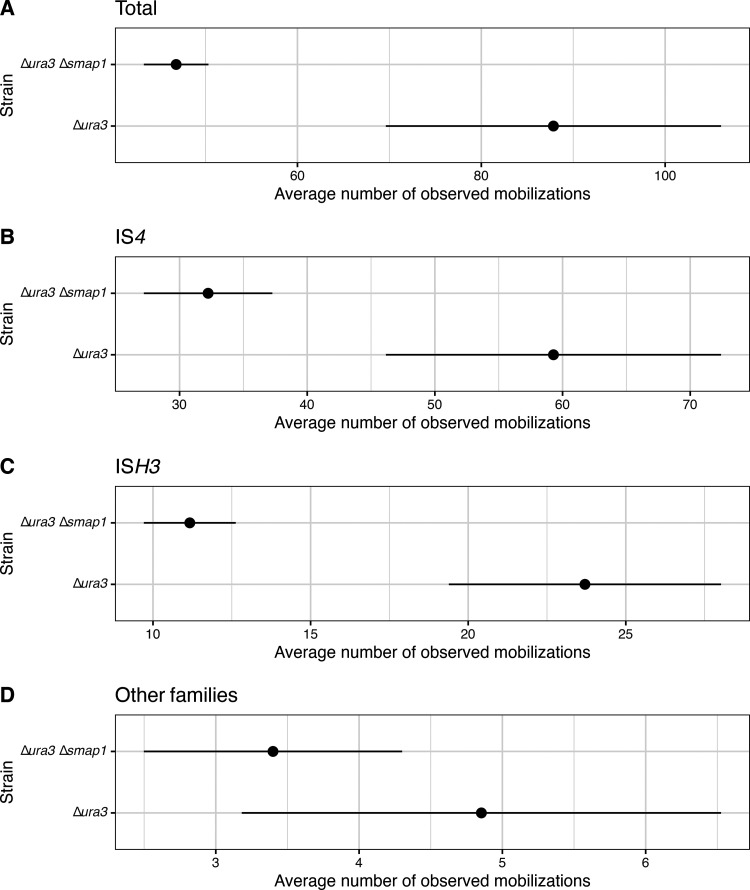
Detected mobilizations for decomposed insertion sequence families. The average normalized number of clusters is shown for each strain. Shown are the results for the pool of all insertion sequences (A), the IS*4* family only (B), the IS*H3* family only (C), and the other families (D). Black lines indicate the ranges of the 68% confidence interval.

Transposases are typically encoded within insertion sequences (ISs), a type of transposable element that is ubiquitous across prokaryotes, and are known to mediate self-mobilization to new locations in the genome (54, 55). The *H. salinarum* NRC-1 mobilome is comprised of 80 full and 33 partial IS elements of eight families (ISfinder/ISbrowser) (56, 57), some of which are known to introduce phenotypic diversity in flotation, by disrupting the *gvp* locus at a 1 to 5% frequency, and also in phototrophic energy production, by disrupting the bacteriorhodopsin gene (*bop*) locus at a 0.01% frequency, potentially driving niche acclimation in brine pools (39, 58, 59). Notably, SmAP1 bound 24 of the 33 mobilome transcripts (enrichment *P* value of 10^−14^) (Fig. 5A and Fig. S1E), consistent with their low GC content (Fig. 5B) and the previously implicated role of its bacterial homolog in regulating transposition events (60, 61). Out of the 33 mobilome proteins, only 4 were detected at the protein level (Fig. 5A and C), including 3 TnpB proteins encoded by IS elements of the IS*200*/IS*605* family subgroup IS*1341* (VNG_0013C, VNG_0044H, and VNG_2652H) and 1 protein encoded by the multicopy IS*H2* element (VNG_0210H) belonging to the IS*H8* family (see Table S3 at https://doi.org/10.6084/m9.figshare.21936396.v2 for IS information). All mobilome proteins except one (VNG_0051a) were present in the SWATH-MS assay library, and none were predicted to be membrane associated. Moreover, they all produced at least one suitable tryptic peptide (≥7 and ≤30 amino acids) when digested *in silico* using Rapid Peptides Generator (62). Notwithstanding their low CAIs (Fig. 5D), the high mRNA abundance (Fig. 5E) and the presence of TPSs suggest that the mobilome proteins were not detected by virtue of being expressed at a low abundance and possibly due to the repression of translation by SmAP1 and asRNAs (Fig. 5A). For instance, the translational repression of VNG_0112H (IS*H3* family) would be consistent with the observed pileup of Ribo-Seq reads at the 5′ end of the transcript, which is colocated with SmAP1-binding sites and a TPS (Fig. S4). Together, these observations suggest that SmAP1 binding might lead to a potentially stalled ribosome-transcript complex, which may then be targeted by an endonuclease in a well-known mechanism called “no-go” decay, as previously hypothesized for similar observations (48). The evidence provided by the atlas offered confidence for further wet-lab experimental exploration. Therefore, we investigated the role of SmAP1 in the regulation of IS element-mediated genome reorganization by performing long-read DNA sequencing (DNA-Seq) to quantify the transposition events for each IS family in a Δ*ura3* Δ*smap1* strain and its parent Δ*ura3* strain (Fig. 6 and Fig. S5; see also Table S4 at https://doi.org/10.6084/m9.figshare.21936396.v2 and Data Set S8 at https://doi.org/10.6084/m9.figshare.21936399.v2). In so doing, we discovered that knocking out SmAP1 significantly decreased the overall number of transposition events (Fig. 6A), particularly the transposition of the IS*4* and IS*H3* families (Fig. 6B and C).

10.1128/msystems.00816-22.4 FIG S4 VNG_0112H, a transposase encoded by the IS*H3B* element. Tracks show various features described on the left-hand side. Green tick marks represent transcript processing sites (TPSs), red rectangles represent SmAP1-binding sites, a blue rectangle (reverse strand) represents the open reading frame for the transposase VNG_0112H, and a green rectangle (reverse strand) represents the IS*H3B* element. Gray single-nucleotide-resolution bar plots represent RNA-Seq and Ribo-Seq coverages. TP2, mid-exponential growth phase. Download FIG S4, TIFF file, 3.5 MB. Copyright © 2023 Lorenzetti et al. 2023 Lorenzetti et al. https://creativecommons.org/licenses/by/4.0/ This content is distributed under the terms of the Creative Commons Attribution 4.0 International license.

10.1128/msystems.00816-22.5 FIG S5 Detected mobilization events. (A) Detected insertions. (B) Detected excisions. Observed events are the numbers of clusters detected for each type of mobilization. All of the cluster types are represented, considering those classified as predominant, common, and rare. Bars are color-coded according to insertion sequence families. Download FIG S5, TIF file, 2.3 MB. Copyright © 2023 Lorenzetti et al. 2023 Lorenzetti et al. https://creativecommons.org/licenses/by/4.0/ This content is distributed under the terms of the Creative Commons Attribution 4.0 International license.

### (ii) Role of post-transcriptional regulation in governing environmental responsiveness and timing of gas vesicle biogenesis.

**FIG 7:**
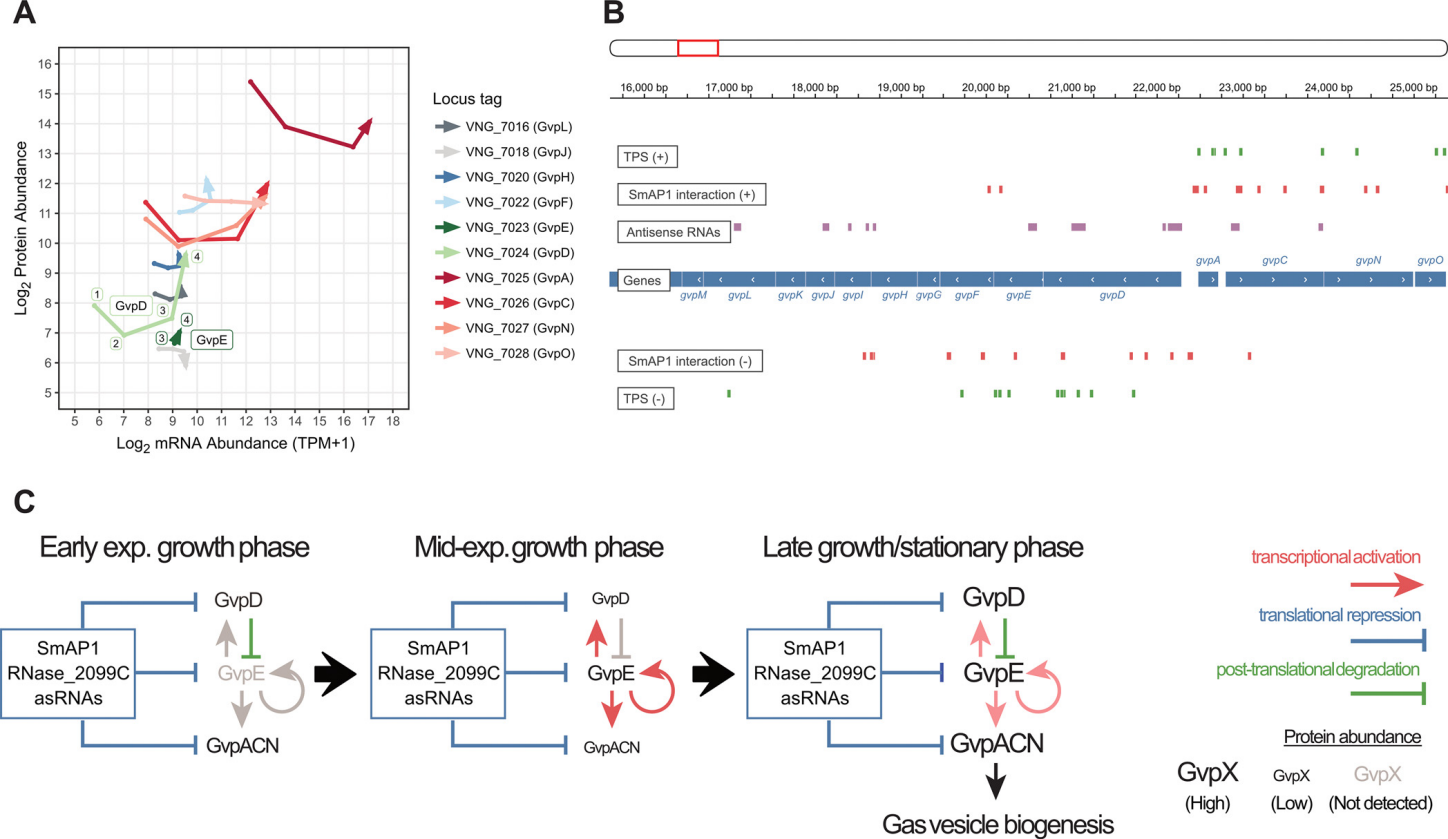
post-transcriptional regulation of *gvp* operons. (A) Arrows representing how each of the gas vesicle operon genes (color-coded) (protein names are in parentheses) behaves regarding its log_2_-transformed protein abundance (*y* axis) and mRNA abundance (*x* axis) across consecutive physiological states (TP1, early exponential growth phase; TP2, mid-exponential growth phase; TP3, late exponential growth phase; TP4, stationary phase). We represent the *gvpMLKJIHGFED* and *gvpACNO* operons, except for a few elements (*gvpG*, *gvpI*, *gvpK*, and *gvpM*) whose protein levels were not detected by our SWATH-MS approach. (B) The genome browser snapshot reveals the regions of *gvpDEFGHIJKLM* (reverse strand) and *gvpACNO* (forward strand) (GenBank accession number NC_001869.1 [bp 16,000 to 25,500]). We depict genes as blue rectangles. Tracks show various features described on the left-hand side. Green ticks represent transcript processing sites (TPSs), red rectangles represent SmAP1-binding sites, and purple rectangles represent annotated antisense RNAs. (C) Time-point-wise regulatory scheme of gas vesicle proteins encoded by the *gvp* cluster. Blue bars represent translational repression, red arrows represent transcriptional activation, and green bars represent posttranslational degradation. Protein abundance is depicted by the font size of the gas vesicle proteins (GvpX).

Gas vesicles are intracellular proteinaceous organelles filled with ambient gas that may be used as buoyancy devices by halophilic archaeal cells to float to the surface to access oxygen, which has poor solubility in hypersaline water (63). The gas vesicles also act in conjunction with sensory rhodopsin-mediated phototaxis to support phototrophic energy transduction by bacteriorhodopsin (64). Hence, the biogenesis of gas vesicles is highly responsive to environmental stimuli, particularly oxygen availability (65). Gas vesicles are made up of two structural proteins: GvpA, a monomer, and GvpC, which wraps around and stabilizes the vesicle assembled from the GvpA polymer (66). Many other proteins (GvpF to -M) are involved in the nucleation and biogenesis of the gas vesicle (67), processes that are regulated by GvpD and GvpE (41). The bidimensional trajectories of the changes in mRNA and protein levels revealed that while the transcript levels of all *gvp* genes, including those encoding the structural proteins, increased across the four growth phases, the corresponding protein levels did not increase until the cells transitioned from the mid-exponential growth phase to the stationary phase (Fig. 7A), which is consistent with the timing of gas vesicle production (68). Together, the multiple levels of evidence in the *H. salinarum* NRC-1 atlas (Fig. 7B and Fig. S6) support a model (Fig. 7C) that explains how the interplay of negative and positive regulation at the transcriptional, post-transcriptional, and translational levels governs the timing and environmental responsiveness of gas vesicle biogenesis.

10.1128/msystems.00816-22.6 FIG S6 Protein-mRNA dynamics and various features of genes encoding gas vesicle biogenesis proteins. We represent the 14 genes comprising the *gvpDEFGHIJKLM* and *gvpACNO* operons in the context of their features. SmAP1 binding, antisense RNAs (asRNAs), and transcript processing sites (TPSs) are enriched in this cluster (*P* = 2.4 × 10^−7^, 3 × 10^−3^, and 3.8 × 10^−2^, respectively). The heat map represents the log_10_-transformed expression profile of proteins (a pseudocount was imputed for missing values), mRNAs (TPM+1), and ribosome-protected mRNA fragments (RPFs) (TPM+1). Heat maps also represent the respective log_2_-transformed translational efficiency (TE) and ribosome occupancy (RO) values for each time point. COG, clusters of orthologous genes; 2099, log_2_ fold change (LFC) of transcripts in the absence of RNase_2099C; CAI, codon adaptation index; TP1, early exponential growth phase; TP2, mid-exponential growth phase; TP3, late exponential growth phase; TP4, stationary phase. Download FIG S6, TIFF file, 2.7 MB. Copyright © 2023 Lorenzetti et al. 2023 Lorenzetti et al. https://creativecommons.org/licenses/by/4.0/ This content is distributed under the terms of the Creative Commons Attribution 4.0 International license.

Based on the absolute abundances and relative changes in mRNA and protein levels, we posit that *gvp* genes were constitutively transcribed across all phases of growth. But the translation of *gvp* transcripts required further transcriptional activation by GvpE (69), which was prevented in the early and mid-exponential growth phases by GvpD. Specifically, in the early growth phase, GvpD was highly abundant and above a threshold at which it drives the degradation of GvpE (70, 71) (Fig. 7A and C). As cells transitioned from the early to the mid-exponential growth phase, SmAP1, RNase_2099C, and asRNAs acted in concert to repress the translation of *gvp* transcripts, which was especially evident in the pileup of ribosomal footprints in the 5′ segment of the *gvpA* transcript. This putative post-transcriptional repression of translation resulted in the growth-associated dilution of the Gvp protein abundance despite a steady increase at the mRNA level (Fig. 7A and C and Fig. S7A). As a consequence, the GvpD protein abundance dropped below the above-mentioned threshold, disrupting its ability to drive the continued degradation of GvpE. This is consistent with the observation that the GvpE protein was detected only in the later stages of growth after the GvpD abundance had decreased (Fig. 7A and C). Moreover, the appearance and subsequent increase in the abundance of GvpE after the mid-exponential growth phase likely resulted in the transcriptional activation of all *gvp* genes (Fig. 7A and C). Indeed, the mRNA levels of all *gvp* genes increased by >4-fold in the mid-exponential growth phase (despite active cell division), unlike the moderate (~2-fold) albeit steady increase observed in the early and late phases of growth (Fig. 7A). The transcriptional activation of all *gvp* genes likely overcame SmAP1-, RNase_2099C-, and asRNA-mediated post-transcriptional repression to upregulate translation via increased ribosomal readthrough (Fig. 7C and Fig. S7A). The resulting dramatic increase in the abundance of the GvpN and GvpO proteins, as well as the chaperone GvpF, potentially triggered the recruitment of GvpA to initiate gas vesicle assembly (67). Concomitantly, in the stationary phase, the GvpD protein level increased above the threshold, likely restoring GvpE degradation, thereby disrupting the transcriptional activation of *gvp* genes and potentially terminating the further translation of gas vesicle proteins (Fig. 7C). So, in essence, the interplay among the GvpD-mediated degradation of GvpE, the transcriptional activation of *gvp* genes by GvpE, and the post-transcriptional repression of the translation of *gvp* genes (likely mediated by SmAP1, asRNAs, and RNase_2099C) together modulated the timing of gas vesicle biogenesis. In this scheme, subtle changes in the interplay across the different levels of regulation could drive the rapid initiation or termination of gas vesicle biogenesis given that the transcripts and the monomeric structural proteins are maintained at relatively high abundances but that the regulatory (GvpD and -E) and some accessory (e.g., GvpJ and -L) proteins are present at significantly lower abundances across all growth phases.

10.1128/msystems.00816-22.7 FIG S7 (A) *gvpACN* loci reveal differential patterns of Ribo-Seq signals. We present the three consecutive loci (VNG_7025 to VNG_7027) comprising the *gvpACN* region (blue rectangles). The time-point-wise Ribo-Seq- and RNA-Seq-normalized profiles are represented by gray bars. Red rectangles represent SmAP1-binding sites, green tick marks represent transcript processing sites (TPSs), and purple rectangles represent antisense RNAs. Each track was automatically scaled using the Autoscale feature of Integrative Genomics Viewer. We observe that pileups of Ribo-Seq emerge after the late exponential growth phase (TP3), indicating that the elongation phase of translation intensifies late on growth. Concurrently, we see SmAP1-binding sites either immediately before or spanning the region where the peaks emerge, indicating the role of this protein as a translational regulator. TP1, early exponential growth phase; TP2, mid-exponential growth phase; TP4, stationary phase. (B) VNG_0042G, a TnpB protein encoded by the IS*H39* element from IS*200*/IS*605* family subgroup IS*1341*. Tracks show various features described on the left-hand side. Green tick marks represent transcript processing sites, red rectangles represent SmAP1-binding sites, a purple rectangle (forward strand) represents an annotated antisense RNA, a blue rectangle (reverse strand) represents the open reading frame for TnpB, and a green rectangle (reverse strand) represents the IS*H39* element. Gray single-nucleotide-resolution bar plots represent RNA-Seq and Ribo-Seq coverages. Download FIG S7, TIF file, 1.1 MB. Copyright © 2023 Lorenzetti et al. 2023 Lorenzetti et al. https://creativecommons.org/licenses/by/4.0/ This content is distributed under the terms of the Creative Commons Attribution 4.0 International license.

## DISCUSSION

This study has uncovered that a strikingly large proportion of protein-coding genes (54%) in the *H. salinarum* NRC-1 genome are potentially post-transcriptionally regulated. Notably, this estimate of the scale of post-transcriptional regulation is based on a compilation of evidence from a limited set of contexts (i.e., primarily under standard growth conditions). It is noteworthy that a comparison of the changes in the absolute and relative abundances of mRNAs and proteins just over batch culture growth provided evidence for the post-transcriptional control of 7% of all protein-coding genes. Importantly, the evidence for the post-transcriptional regulation of 7% of these genes was based on two very stringent requirements, that (i) the mRNA levels were in the top quintile and the protein levels were in the bottom quintile or null in a given physiological state or (ii) the mRNAs were at least 2-fold upregulated, with the corresponding protein levels being at least 2-fold downregulated in a given physiological state transition. These stringent cutoffs were selected to identify high-confidence evidence of post-transcriptional regulation, which could have led to a significant underestimation of the actual number of genes that were truly subject to post-transcriptional regulation. Needless to say, lowering these thresholds would likely uncover evidence that a significantly larger number of genes were post-transcriptionally regulated over the four phases of growth in batch culture. Furthermore, different sets of genes were previously reported to have a discordant relationship between mRNA and protein levels in other environmental contexts such as shifts in oxygen tension (45) and exposure to gamma irradiation (46). In response to gamma irradiation, 47 upregulated transcripts had a direction of change incompatible with that of their respective proteins. Of those, only 5 are included in the set of 188 putative post-transcriptionally regulated genes identified in the present study. Together, these observations illustrate the importance of the environmental context for characterizing the genome-wide implications of post-transcriptional regulation. Similarly, we have surveyed just three mechanisms (SmAP1, asRNAs, and one RNase) that provide a likely mechanistic explanation for the post-transcriptional regulation of 430 out of 966 transcripts (45%) with TPSs. This suggests that the 536 remaining TPS-associated transcripts are potentially post-transcriptionally regulated by other mechanisms, including endoribonucleases, *trans*-acting antisense RNAs, and small regulatory RNAs (sRNAs), that were not surveyed in this study, although previous work suggested a limited role of *trans*-acting antisense RNAs and sRNAs in archaeal regulation (72). Furthermore, we attempted to generate knockout mutants for all RNases, but many appeared to be essential as we could not establish the strains after multiple attempts. While we were successful in knocking out three other nonessential RNases (VNG_1503C, VNG_2512G, and VNG_2647G), we did not pursue these strains for further in-depth investigation as the knockouts did not result in any observable phenotypic consequences under standard laboratory growth conditions (21). For this reason, we decided to focus on just RNase_2099, for which transcriptome data were reported previously. Nonetheless, we can expect that some or all of these antisense RNAs, sRNAs, and RNases might indeed post-transcriptionally regulate many more genes in the *H. salinarum* NRC-1 genome, especially in ecological contexts that require rapid physiological state transitions for environmental acclimation.

Transcriptome-wide binding analysis by RIP-Seq implied a global role for SmAP1 in the post-transcriptional regulation of at least 397 genes. The validation of post-transcriptional regulation by SmAP1 with independent data sets, including colocalization with TPSs and discordance between mRNA and protein levels, is essential to rule out spurious binding, especially binding to high-abundance transcripts, that might have resulted from the overexpression of SmAP1 to perform RIP-Seq. It is noteworthy in that regard that some spurious binding events were ruled out by including RIP-Seq using just the overexpressed epitope tag as a negative control. Notwithstanding that caveat, our results were biologically meaningful and consistent with those of previous reports, such as the finding that the action of SmAP1 in *H. salinarum* NRC-1 appears to have mechanistic similarity to those of its counterparts in other archaea and Hfq in bacteria, such as preferentially targeting AU-rich sequences and regulating itself (36). Autoregulation by the bacterial ortholog of SmAP1, Hfq, has also been reported previously in Escherichia coli (73, 74) and Sinorhizobium meliloti (75). By reviewing RIP-Seq results from studies of other archaea, we discovered that SmAP1 also binds to its own transcript in S. solfataricus (SSO6454) (34). The absence of evidence of the autoregulation of SmAP1 in H. volcanii (32) is likely a technical artifact because the microarray used for RIP-chromatin immunoprecipitation (ChIP) interrogated binding to only noncoding RNAs and did not include probes for coding genes, including the SmAP1 coding sequence (HVO_2723). Furthermore, the genes targeted by SmAP1 also bear functional similarity to other organisms wherein SmAP1 has been implicated in the regulation of motility (33, 76) and its ortholog has been implicated in the regulation of transposition (60, 61). Notably, of the 33 nonredundant mobilome proteins (see Table S3 at https://doi.org/10.6084/m9.figshare.21936396.v2) with above-average mRNA levels, only 4 were detected by SWATH-MS in this study, suggesting that they were all post-transcriptionally repressed. By analyzing proteomics data from PeptideAtlas (77, 78) and PRIDE (79), including data under ProteomeXchange identifiers PXD003667 (80) and PXD015192 (81), we confirmed that 50% of the 33 mobilome proteins have been previously detected, depending on the techniques and biological conditions. In addition, except for VNG_0051a, we established that these proteins bear the features required for detection by SWATH-MS. With that reasoning, we posit that the lack of detection of transposases in this study is due to their low abundance or complete absence. Together, these findings make a compelling case that the translation of IS element-encoded transposases, and, therefore, the transposition of mobile genetic elements, is post-transcriptionally regulated. Translational inhibition of transposases might have evolved as a fail-safe measure to prevent transposition in most contexts and allow their rapid activation in stressful environmental contexts wherein the benefits of genome reorganization could outweigh their deleterious effects (82).

Notwithstanding their mechanistic and functional similarities with counterparts in other archaea and even bacteria, we discovered that the consequences of the SmAP1-mediated regulation of transposition by some families of IS elements in *H. salinarum* NRC-1 are counterintuitive. Specifically, while we had expected that SmAP1 likely represses the translation of transposase transcripts, to our surprise, we discovered that the deletion of SmAP1 resulted in a decreased frequency of transposition by IS elements of the IS*4* and IS*H3* families, which brought to the forefront two outstanding questions. First, in addition to directing targeted post-transcriptional processing and repression of transcripts, (how) does SmAP1 also mediate transposition by IS elements? Second, despite targeting AU-rich sequences, how do SmAP1 and its counterparts accomplish the regulation of specific subsets of target genes in a context-specific manner? While the first question will need further investigations into the mechanisms of SmAP1 action on transposition events, our integrated analysis has provided some clues to address the second question, such as evidence that SmAP1 might act in concert with other post-transcriptional regulatory mechanisms, *viz.*, asRNAs and RNase_2099C, to gain specificity for transcripts. So while SmAP1 appears to be expressed constitutively and maintained at a median abundance (Fig. S1D), its mode and target of action may be governed by other factors, such as the conditional expression of asRNAs, which could guide SmAP1 action on specific transcripts in a manner similar to that of its bacterial counterpart (25). Indeed, in H. volcanii, the global oxidative stress response upregulates asRNAs, with the consequential downregulation of specific transposase mRNAs, especially those of the IS*4* family (72). For example, SmAP1 and an asRNA may jointly regulate transposition events by binding to the 5′ end of the TnpB (VNG_0042G) transcript to repress the translation of this putative RNA-guided endonuclease, which is encoded by IS*H39* (IS*200*/IS*605* family) and possibly part of the transposition apparatus (see Fig. S7B in the supplemental material) (83, 84). Thus, the SmAP1-mediated post-transcriptional regulation of mobile elements appears to have pleiotropic consequences depending on the IS family, with a repressive role for IS*200*/IS*605*, as reported previously for Salmonella enterica (61), and an enhancing role for IS*H3* and IS*4*. Indeed, SmAP1 might facilitate the translation of transcripts, considering its hairpin-melting potential (85) and its role as a recruiter for translational complex subunits (86).

The current study has revealed the extensive interplay of post-transcriptional regulation with regulation at other levels of information processing, which may mediate rapid adaptive responses to environmental changes (e.g., genome reorganization by triggering the transposition of IS elements and vertical relocation by activating gas vesicle biogenesis). In the case of gas vesicle biogenesis, we observed that the high abundance and relative increase in transcript levels of the gas vesicle structural genes did not manifest in increased protein levels until the repression of translation was overcome in later stages of growth, which is associated with stressful conditions, including anoxia and nutrient limitation. Previously, we demonstrated that RNase_2099C is transcriptionally coregulated with genes of the aerobic physiological state but acts on transcripts of the anaerobic state (21). In this arrangement, the interplay of RNase_2099C with transcriptional regulation generates an efficient state transition switch. For instance, the RNase_2099C-mediated repression of positive transcriptional autoregulation enables the rapid shutdown of ATP-consuming K^+^ uptake to conserve energy under anoxic conditions with high potassium availability. Gas vesicle biogenesis (response to light and oxygen) appears to be regulated in a similar setup albeit with an expanded set of players. Specifically, the interplay of the GvpD-mediated degradation of GvpE, the GvpE-mediated transcriptional activation of *gvp* genes, and the post-transcriptional repression of gas vesicle protein synthesis through the potential interplay of SmAP1, RNase_2099C, and asRNAs is likely critical for mediating the rapid initiation and termination of gas vesicle biogenesis. The genome-wide atlas reveals that a large proportion of genes in the *H. salinarum* NRC-1 genome are likely subject to such post-transcriptional regulation, and as such, it will serve as an interactive hypothesis generator to drive the in-depth characterization of specific mechanisms of rapid environmental acclimation.

## MATERIALS AND METHODS

### Strains, media, and growth conditions.

We grew Halobacterium salinarum NRC-1 in complex medium (CM) (250 g/L NaCl, 20 g/L MgSO_4_·7H_2_O, 3 g/L sodium citrate, 2 g/L KCl, and 10 g/L bacteriological peptone). Δ*ura3* and Δ*ura3* Δ*smap1* mutant strains had their media supplemented with uracil (50 μg/mL). Vector-harboring strains wtp-pMTF-cMyc and wtp-pMTF-SmAP1-cMyc had their media supplemented with mevinolin (20 μg/mL). All of the cultures were grown at 37°C under light, with constant agitation at 125 rpm (unless otherwise specified). For cloning steps, we used Escherichia coli DH5α grown in lysogeny broth (LB) (10 g/L tryptone, 5 g/L yeast extract, 10 g/L NaCl [pH 7.5]) at 37°C under constant agitation. Carbenicillin (50 μg/mL) was added to LB when necessary.

### Construction of an SmAP1 knockout strain and a cMyc-tagged SmAP1-expressing strain.

The SmAP1 knockout strain (Δ*ura3* Δ*smap1* [Δ*VNG_1673G* Δ*VNG_1496G*]) was constructed from a parent Δ*ura3* strain (Δ*VNG_1673G*) by using the pop-in/pop-out method with two-step selection by mevinolin and 5-fluoroorotic acid (5-FOA) (87). PCR was used to confirm the genotype of null mutants selected by 5-FOA (see Table S5 at https://doi.org/10.6084/m9.figshare.21936396.v2). We evaluated the growth curve phenotype (see Fig. S8 in the supplemental material) by culturing strains in CM supplemented with uracil (50 μg/mL) at 37°C at 125 rpm.

10.1128/msystems.00816-22.8 FIG S8 Growth curves of the Δ*ura3* and Δ*ura3* Δ*smap1* strains. We conducted a growth curve experiment with three biological replicates for the Δ*ura3* (blue lines) and Δ*ura3* Δ*smap1* (orange lines) strains. Line types depict each of the biological replicates. Download FIG S8, TIF file, 0.8 MB. Copyright © 2023 Lorenzetti et al. 2023 Lorenzetti et al. https://creativecommons.org/licenses/by/4.0/ This content is distributed under the terms of the Creative Commons Attribution 4.0 International license.

To create the recombinant SmAP1-cMyc protein, we used the pMTF-cMyc vector (4). The SmAP1-encoding gene (VNG_1496G) was amplified (see Table S5 at https://doi.org/10.6084/m9.figshare.21936396.v2) and purified using QIAquick PCR purification (Qiagen). The amplification product was cloned into the vector pMTF-cMyc upstream of the region encoding the 13-cMyc tag. The procedure was carried out by digesting pMTF-cMyc with endonucleases NdeI and BamHI (Fermentas), with further ligation of the *smap1* amplicon by T4 DNA ligase (Fermentas). The clone was transformed into E. coli DH5α and confirmed by PCR and Sanger sequencing. Vectors were extracted and transformed into the *H. salinarum* NRC-1 strain to create strains wtp-pMTF-SmAP1-cMyc (SmAP1-cMyc overexpression) and wtp-pMTF-cMyc (cMyc overexpression).

### SmAP1-RNA coimmunoprecipitation.

*H. salinarum* strains wtp-pMTF-SmAP1-cMyc and wtp-pMTF-cMyc were grown until they reached an optical density at 600 nm (OD_600_) of ~0.75. We centrifuged 20 mL of the cell culture at 3,700 relative centrifugal force (RCF) for 10 min and resuspended the cells in 12 mL of a basal solution (CM without bacteriological peptone). The cellular suspension solution was transferred to Petri dishes on ice and submitted to 800 × 100 μJ/cm^2^ UV radiation inside a UVC 500 cross-linker (Amersham Biosciences). The solution was carefully transferred to 50-mL tubes and centrifuged at 3,700 RCF for 15 min at 4°C. Cells were resuspended in 1 mL of a lysis solution (1× phosphate-buffered saline [PBS], 0.1% sodium dodecyl sulfate [SDS], 0.5% deoxycholate, 0.5% NP-40, proteinase inhibitor [1 tablet for 100 mL] [catalog number S8830; Sigma], RNaseOUT inhibitor [2 μL/10 mL] [Invitrogen]) on ice and incubated for 5 min. The suspension was centrifuged at 10,000 RCF for 5 min at 4°C. The supernatant was separated and incubated with 10 μL of Dynabeads M-450 anti-mouse IgG (catalog number 11041; Invitrogen) for 10 min at 4°C to remove spurious interactions. After incubation, the solution was centrifuged at 10,000 RCF for 5 min at 4°C. The supernatant was incubated overnight, under constant agitation, at 4°C with 60 μL of anti-cMyc antibody-coated beads (catalog number M4439; Sigma). The beads were immobilized using a magnetic rack, washed twice using 1 mL of a lysis solution followed by two rounds of washing with 1 mL of a saline solution (5× PBS, 0.1% SDS, 0.5% deoxycholate, 0.5% NP-40), and finally washed with 1 mL of Tris-EDTA (TE) buffer. The beads were resuspended in 100 μL of TE and incubated at 65°C for 10 min. The suspension was centrifuged at 14,000 RCF for 30 min at 25°C. We added 120 μL of TE-SDS (0.1% SDS) to the supernatant and incubated it for 30 min at 65°C. Two aliquots were separated: one for the Western blot assay and another for RNA isolation prior to sequencing.

### SmAP1-cMyc Western blot assay.

We verified the presence of the SmAP1 protein in the coimmunoprecipitated samples using the Western blot assay. Aliquots of sample buffer (30% [vol/vol] glycerol, 9.2% [wt/vol] SDS, 1% [wt/vol] bromophenol blue, 20% [vol/vol] β-mercaptoethanol, 0.25 M Tris-HCl [pH 7.0]) were added, and the samples were denatured at 95°C for 5 min. Denatured samples (20 μL) were submitted to SDS–10% polyacrylamide gel electrophoresis (SDS-PAGE). A PageRuler prestained protein ladder (Fermentas) was used as the weight marker and transference control. Gel and Hybond ECL nitrocellulose membranes (GE) were dipped in transfer buffer for 10 min.

Membrane transfer was performed at 100 V for 1 h. The membrane was washed with PBS–0.1% (vol/vol) Tween 20 (PBS-T) and incubated in PBS-T with milk at room temperature for 1 h. After the blocking step, the membrane was quickly washed twice with PBS-T. The primary antibody (anti-cMyc) was diluted (1:3,000) in PBS-T, and incubation was carried out at 4°C under constant agitation overnight. The membrane was rewashed with PBS-T and incubated in PBS-T at room temperature under constant agitation for 15 min. The secondary antibody (anti-mouse IgG-peroxidase, catalog number A4416; Sigma) was diluted (1:3,000) in PBS-T, and incubation was carried out at room temperature under constant agitation for 1 h. The membrane was quickly washed twice using PBS-T and incubated in PBS-T at room temperature under constant agitation for 15 min. We used the ECL Western blotting detection reagents (GE) to develop the membrane, and images were obtained using the ChemiDoc XRS+ system (Bio-Rad).

### SmAP1 RIP-Seq and data analysis.

The coimmunoprecipitated RNA samples were subjected to protein digestion using proteinase K (Fermentas) and purified using the MinElute reaction cleanup kit (Qiagen) with a DNase treatment step. We quantified the RNAs in the samples using a Quant-iT RiboGreen RNA assay (Invitrogen) and prepared them for sequencing using the TruSeq mRNA stranded kit (Illumina). Before sequencing, to equalize the concentrations, quantification was performed by using the Kapa Library Quant kit (Kapa Biosystems). Samples were sequenced using the MiSeq Reagent v2 kit (Illumina) for 50 cycles, using the single-end mode, in a MiSeq instrument (Illumina).

We processed the sequenced libraries using the ripper pipeline (see Table S6 at https://doi.org/10.6084/m9.figshare.21936396.v2) to obtain putative SmAP1-binding regions. Briefly, the software (i) trims the low-quality ends and adapters from reads using Trimmomatic (88); (ii) aligns trimmed reads to the reference genome (NCBI assembly ASM680v1) using HISAT2 (89), without gaps, splicing, or soft clipping; (iii) converts alignment files from SAM format to BAM format using SAMtools (90); (iv) adjusts multimapping reads using MMR (91); (v) computes single-nucleotide-resolution transcriptome signals using BEDTools (92); and (vi) computes a coordinate-wise log_2_ fold change (LFC) for coimmunoprecipitated samples relative to control samples and identifies regions with at least 10 consecutive nucleotides satisfying a log_2_ fold change of ≥1. Interaction regions for two biological replicates (BR1 and BR2) were merged since their intersection of SmAP1-bound genes had a 3.8-fold enrichment over the expected value (observed, 157 genes; expected, 41.44 genes; *P* = 3.14 × 10^−71^). We tested the fold enrichment significance by using the SuperExactTest::MSET function (93).

### Preparation and acquisition of proteomics samples.

Sample preparation and data acquisition for the time course measurements of the *H. salinarum* proteome were performed as described by Kusebauch et al. (unpublished). In brief, *H. salinarum* NRC-1 was cultured in CM. Cultures were grown in triplicate (37°C with shaking at 220 rpm) and illuminated (~20 μmol/m^2^/s) in Innova 9400 incubators (New Brunswick). Cultures were harvested at four time points: early exponential phase (OD_600_ = 0.2; 14.3 h), mid-exponential phase (OD_600_ = 0.5; 21.5 h), late exponential phase (OD_600_ = 0.8; 28.8 h), and stationary phase (40.8 h). Cells were collected by centrifugation (8,000 × *g* for 2 min at 4°C). Cell pellets were resuspended in MilliQ water and disrupted at 4°C using ceramic beads (Mo Bio Laboratories) and a Precellys 24 homogenizer (Bertin Corp.). The protein content was determined by a bicinchoninic acid (BCA) assay (Thermo Fisher). Proteins were reduced (5 mM dithiothreitol [DTT] [45 min at 37°C]), alkylated (14 mM iodoacetamide [30 min at room temperature in the dark]), and digested with trypsin (1:50 enzyme-to-substrate ratio [37°C for 16 h]). Samples were desalted with tC_18_ Sep-Pak cartridges (Waters). Sample analysis was performed on a TripleTOF 5600+ system equipped with a Nanospray-III source (Sciex) and an Eksigent Ekspert nanoLC 425 with cHiPLC system in trap-elute mode (Sciex). Peptides were separated with a gradient from 3% to 33% of 0.1% (vol/vol) formic acid in acetonitrile for 120 min. Data were collected in MS/MS^ALL^ SWATH acquisition mode using 100 variable acquisition windows.

### SWATH-MS data analysis.

SWATH-MS data were analyzed using Spectronaut software (version 15.5.211111.50606) and an assay library for *H. salinarum* NRC-1 reported by Kusebauch et al. (unpublished). SWATH .wiff raw data files were converted to HTRMS files with the Spectronaut HTRMS converter (version 15.5.211111.50606). Data extraction mass tolerance (MS1 and MS2) was set to dynamic with a correction factor of 1. The dynamic extracted ion chromatogram (XIC) retention time (RT) window was enabled with a correction factor of 1 and local (nonlinear) RT regression. Decoy assays were dynamically generated using the scrambled decoy method, and the library size fraction was set to 1. Identification was performed using the normal distribution estimator with precursor identification results with a *q* value (false discovery rate [FDR]) of <0.1 and protein identification results with a *q* value (FDR) of <0.01. Quantification was performed with interference correction enabled, MS2 ion peak areas of quantified peptides were summed to estimate protein peak areas, and area as the quantity type was selected. The identified precursor quantities were normalized using the Spectronaut built-in global normalization function (median). The four time points in this study were defined as four conditions in the condition setup. We used Spectronaut’s protein quantification and proDA (94) to perform differential expression analysis of proteins. We computed the contrasts of interest and set up a |log_2_ fold change| of ≥1 and an adjusted *P* value of <0.05 as the criteria to determine differentially expressed proteins.

### Nonredundant reference transcriptome.

Many annotation efforts for *H. salinarum* NRC-1 have been made available since the publication of its genome assembly (50). Consequently, cross-referencing findings from publications using different sources has become a challenging and time-consuming task. Moreover, the genome presents redundancy in terms of (quasi)identical paralogs, with most of them being found within minichromosome (pNRC100 and pNRC200) repetitive regions (95) and contained within multicopy insertion sequences (96). To solve the problems of the annotation multiplicity and gene redundancy, we extracted coding and noncoding sequences (tRNAs, rRNAs, signal recognition particle RNA, and RNase P) from different annotation sources for the *H. salinarum* NRC-1 and R1 strains (see Table S1 at https://doi.org/10.6084/m9.figshare.21936396.v2) and clustered them using CD-HIT (97). Coding and noncoding genes with at least 95% and 99% global amino acid and nucleotide identities, respectively, were grouped and represented by a single entity anchored by the sequence and locus tag given by the latest large-scale annotation effort for *H. salinarum* NRC-1 (51). We considered only sequences represented in this annotation. We also collected and parsed clusters of orthologous genes (COGs) (98) to functionally categorize the nonredundant reference transcriptome and classified insertion sequence families using the ISfinder (57) and ISsaga (99) platforms. The code to reproduce this annotation simplification effort is available on GitHub (see halo_nr_tx in Table S6 at https://doi.org/10.6084/m9.figshare.21936396.v2).

### Transcriptome analysis.

We retrieved RNA-Seq and Ribo-Seq data for an *H. salinarum* growth curve experiment available at the NCBI Sequence Read Archive (SRA) under BioProject accession number PRJNA413990 (43). The samples are the same as the ones for which the proteome data were generated, as explained above. We quantified all of the RNA-Seq libraries by mapping them against the *H. salinarum* NRC-1 nonredundant reference transcriptome using kallisto (100), facilitated by the use of the runKallisto pipeline (see Table S6 at https://doi.org/10.6084/m9.figshare.21936396.v2). We performed differential expression analysis for the RNA-Seq and Ribo-Seq time course experiments (43) using DESeq2 (101). Only genes satisfying a |log_2_ fold change| of ≥1 and an adjusted *P* value of <0.05 were considered differentially expressed. We generated the transcriptome coverage signal for genome browsing using the frtc pipeline (102) (see Table S6 at https://doi.org/10.6084/m9.figshare.21936396.v2). Briefly, the tool trims reads using Trimmomatic (88), aligns them to the reference genome (NCBI assembly ASM680v1) using HISAT2 without splicing (89), adjusts multimapping instances using MMR (91), and computes the genome-wide coverage using deepTools2 (103).

We performed differential expression analysis of the Δ*RNase_2099C* strain by reanalyzing data reported previously (21), deposited in the Gene Expression Omnibus (GEO) under accession number GSE45988. Briefly, we used limma (104) to process the data and computed the Δ*RNase_2099C*-versus-Δ*ura3* contrast controlling for the growth curve time point effect. We used only data for the mid-exponential (OD_600_ of ~0.4) and late exponential (OD_600_ of ~0.8) growth phases. Only genes satisfying a |log_2_ fold change| of ≥1 and a *P* value of <0.05 were considered differentially expressed.

### Inference of putative post-transcriptionally regulated genes.

We relied on transcriptome and proteome quantitation to infer putative post-transcriptionally regulated genes. For that, we developed two methods: (i) the absolute-abundance-based approach, in which we identified genes producing simultaneously high mRNA levels (transcripts per million [TPM] in the upper quintile) and low protein abundances (lower quintile or undetected), and (ii) the relative-abundance-based approach, in which we inspected differentially expressed genes in physiological state transitions with mRNA levels being upregulated and protein levels being downregulated.

We further inspected the genes identified by the absolute-abundance-based approach, whose proteins were not detected, to remove entries that were likely missed due to technical limitations. After manual inspection, we removed potential transmembrane proteins (as these are difficult to detect), proteins not represented in the assay library due to the lack of suitable peptides for detection by SWATH-MS (e.g., hydrophobicity and peptide length), and proteins not represented in the assay library due to differences in annotation versions. For a protein to be considered a transmembrane protein, we first conducted transmembrane domain prediction for all of the entries encoded by the nonredundant transcriptome using the TOPCONS Web server (105). We manually inspected the results and evaluated the “consensus prediction probability” of transmembrane regions. We required proteins to have at least one transmembrane domain with a considerable extension satisfying a probability of ≥0.9. To aid our judgment, we also pondered empirical evidence (106, 107) and functional annotations. This approach identified 117 genes with expressive mRNA and undetected proteins with a high likelihood of being post-transcriptionally regulated (see Data Set S3 at https://doi.org/10.6084/m9.figshare.21936399.v2).

### Long-read DNA sequencing and analysis.

*H. salinarum* Δ*ura3* and Δ*ura3* Δ*smap1* strains were grown in CM supplemented with uracil until the OD_600_ reached ~0.5. Aliquots of 2 mL of cell cultures were submitted to DNA extraction using a DNeasy blood and tissue kit (Qiagen). DNA samples were quality checked and genotyped using PCR to confirm strains (see Table S5 at https://doi.org/10.6084/m9.figshare.21936396.v2). We prepared the samples for long-read DNA sequencing using the MinION platform (Oxford Nanopore Technologies [ONT]). Libraries were prepared using the SQK-LSK108 kit (ONT) combined with the EXP-NBD103 kit (ONT) to allow multiplexing. The experiment was run using the MinION Mk1B system (ONT) with a FLO-MIN106 flow cell (ONT) for 48 h. Raw data were demultiplexed using Deepbinner (108) and base called using Guppy (ONT). Quality checking was done using Filtlong (see Table S6 at https://doi.org/10.6084/m9.figshare.21936396.v2), and adapter trimming was performed using Porechop (see Table S6 at https://doi.org/10.6084/m9.figshare.21936396.v2).

We used NGMLR (109) to align reads to a modified version of the reference genome, accounting for plasmid long duplications within the pNRC100 and pNRC200 minichromosomes only once (GenBank accession numbers NC_002607.1 [bp 1 to 2,014,239], NC_001869.1 [bp 1 to 150,252], and NC_002608.1 [bp 112,796 to 332,792]). To identify low-complexity structural variations (SVs), the alignments were processed with Sniffles (109), and the VCF files were filtered to keep only insertions and deletions. The sequences of the detected SVs were compared to *H. salinarum* NRC-1 annotated insertion sequences using BLAST (110). Insertions and excisions were annotated only if they satisfied the threshold of at least 75% identity and 80% coverage considering both the query and the subject. These criteria were based on the 80-80-80 rule proposed previously (111) but slightly loosened because of the intrinsically high Nanopore error rates.

We applied a clustering approach for neighboring elements to avoid overestimating the number of identified SVs. SVs of the same class (insertion or excision), caused by the same element, and starting within a 50-bp distance from each other were combined into a single cluster having a mean start point and a support index based on the number of occurrences. Dividing this number of occurrences (*e*) by the local read coverage (25-nucleotide bidirectional flank) (*c*) allowed us to classify SV clusters into three categories: (i) when *e/c* is ≤0.1, the cluster is defined as relatively rare in the population; (ii) when 0.1 < *e/c *≤ 0.5, it is common; and (iii) when *e/c* is >0.5, the cluster is characterized as predominant, indicating that this SV might be fixed in the population genomes.

We computed the total number of clusters of insertions and excisions for each of the libraries and added them up before normalizing the values based on each sample’s total number of aligned reads. To normalize, we identified the library with the highest number of aligned reads and adjusted the others to be comparable. The mean values for normalized counts were computed for both the Δ*ura3* Δ*smap1* and Δ*ura3* strains and compared using a confidence interval of 68% (see Table S6 at https://doi.org/10.6084/m9.figshare.21936396.v2 for code).

### Enrichment analysis and average comparison.

To detect enriched features (e.g., SmAP1 binding, antisense RNAs [asRNAs], and transcript processing sites [TPSs]) within groups of genes, we performed enrichment analysis using the hypergeometric test from R software (stats::phyper function). To compare the averages of features (e.g., half-lives, codon adaptation indices [CAIs], GC [guanine-cytosine] contents, and Δ*RNase_2099C* LFCs) between groups of genes, we used the nonparametric Mann-Whitney U test from R software (stats::wilcox.test function). The significance cutoff of our choice for both statistical tests was a *P* value of <0.05.

### Data collection from miscellaneous sources.

We gathered and parsed data from several sources. We collected asRNA data reported previously by de Almeida et al. (see Table S4 in reference 47). We obtained TPSs from data reported previously by Ibrahim et al. (see Table S1 in reference 48). Redundancy was removed by collapsing asRNAs and TPSs of identical and quasi-identical transcripts. We obtained half-lives from a previously reported microarray experiment (44). Redundancy was removed by computing the average half-lives of identical and quasi-identical genes. We computed the CAI (112) using the coRdon::CAI function (see coRdon in Table S6 at https://doi.org/10.6084/m9.figshare.21936396.v2), taking as the input the 5% most abundant proteins according to our proteomics approach. We computed the GC content using the Biostrings::letterFrequency function.

### *H. salinarum* NRC-1 multi-omics atlas portal.

We developed the *H. salinarum* NRC-1 multi-omics atlas portal by integrating existing components into new resources. Legacy data are stored in an SBEAMS MS SQL server database, which supplements the main MySQL database. A Web service application programming interface (API) implemented in Python and Flask provides uniform access to these resources. We implemented the Web-based user interface using the JavaScript framework Vue.js (see Table S6 at https://doi.org/10.6084/m9.figshare.21936396.v2 for code). We built the heat map interface with the help of the InteractiveComplexHeatmap (113), ComplexHeatmap (114), and Shiny R packages. We built the genome browser by using igv.js (115). The data used to generate the heat maps were prepared as described above, with an additional step for scale adjustment to allow a graphical representation of disparate multimodal omics sources. The quantile-normalized data are also available along with the nonnormalized data (Data Set S1). The Web portal is available at http://halodata.systemsbiology.net.

### Data availability.

SmAP1 RIP-Seq raw data (FASTQ format) and DNA-Seq data (demultiplexed, base called, and trimmed) (FASTQ format) were deposited in the NCBI Sequence Read Archive and are publicly available under BioProject accession number PRJNA808788. Raw DNA-Seq data (FAST5 format) are available at Zenodo (accession number 6303948 [https://doi.org/10.5281/zenodo.6303948]). Supplemental material is available on Figshare (https://doi.org/10.6084/m9.figshare.c.6395322.v3). The code used in this study is available on GitHub in multiple repositories (see Table S6 at https://doi.org/10.6084/m9.figshare.21936396.v2 for links and descriptions).
