# Supplementary figures and images for: A Genome-Scale Atlas Reveals Complex Interplay of Transcription and Translation in an Archaeon
Source: mSystems. 2023 Mar 13;8(2):e00816-22. doi: 10.1128/msystems.00816-22 (PMC10134880; doi:10.1128/msystems.00816-22)

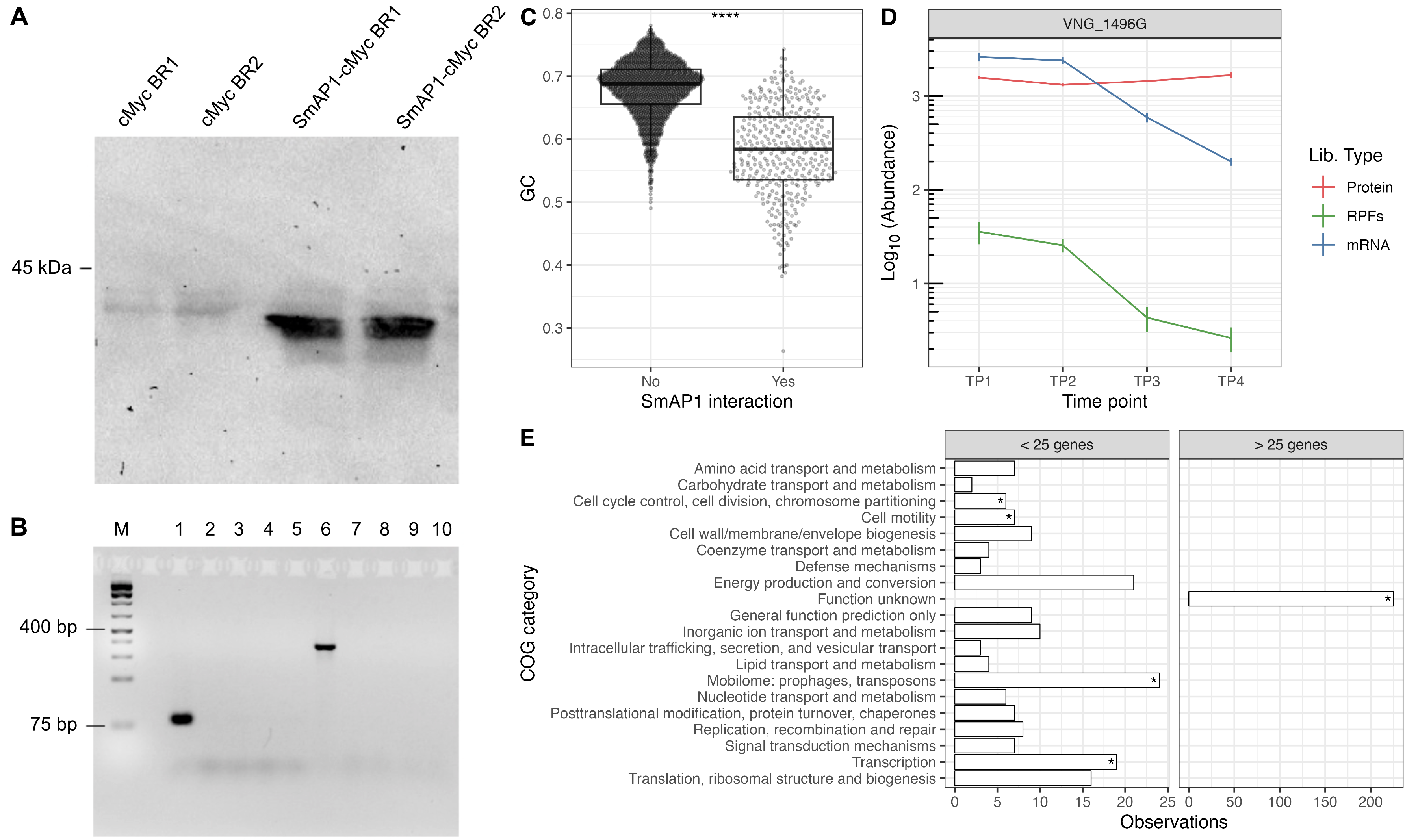

Supplement: FIG S1 [file msystems.00816-22-s0001.tif]

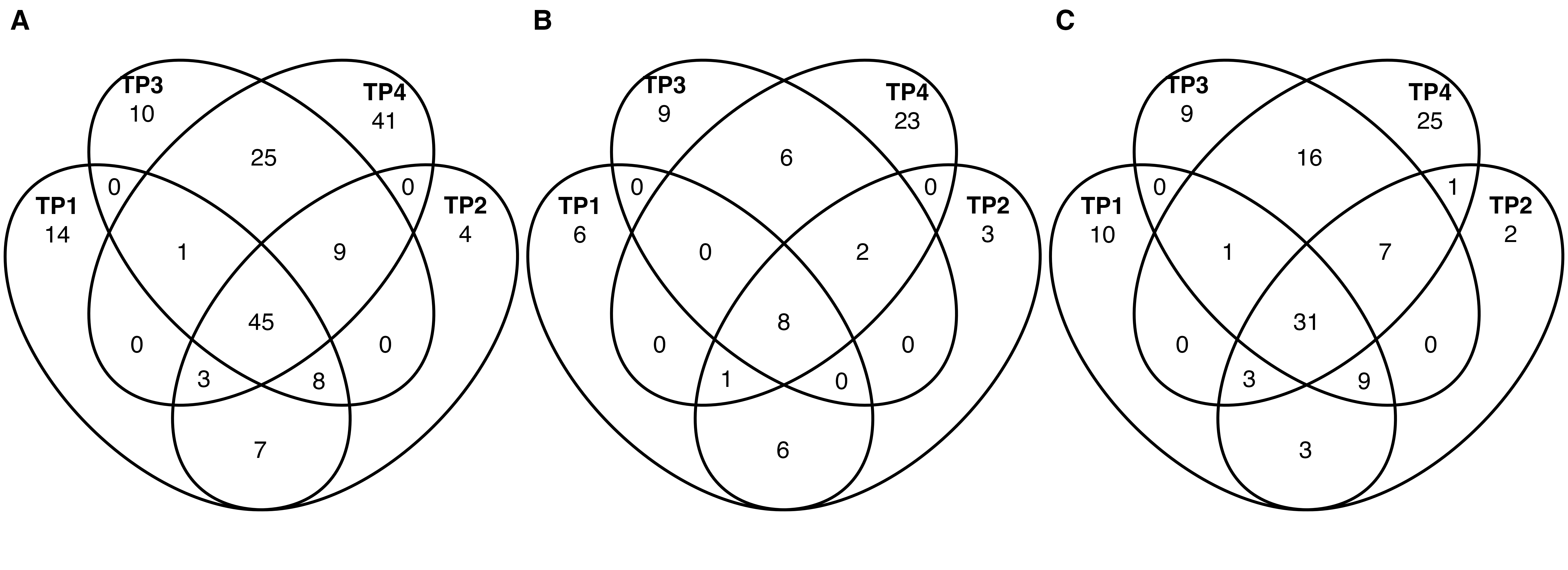

Supplement: FIG S2 [file msystems.00816-22-s0002.tif]

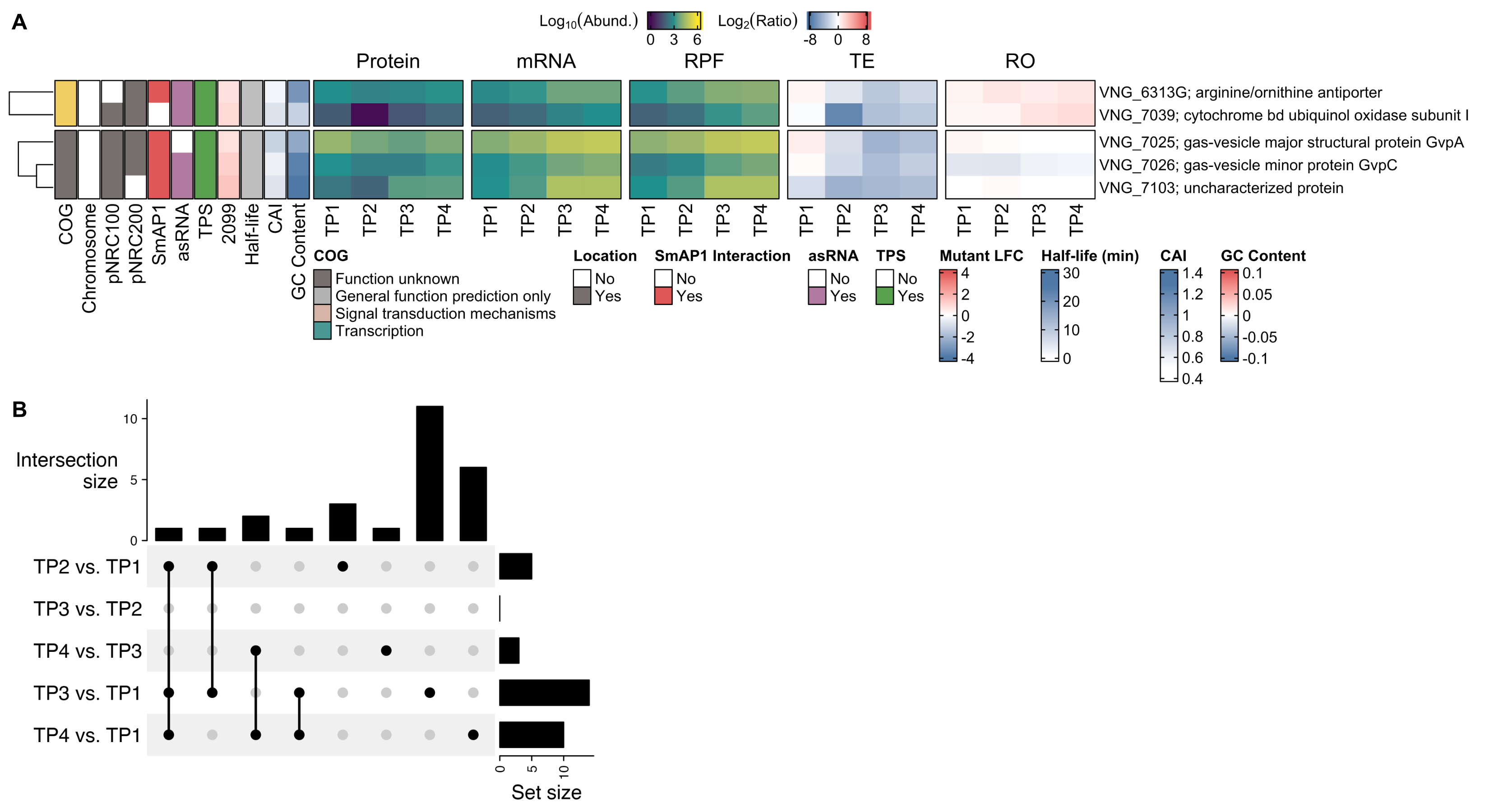

Supplement: FIG S3 [file msystems.00816-22-s0003.tif]

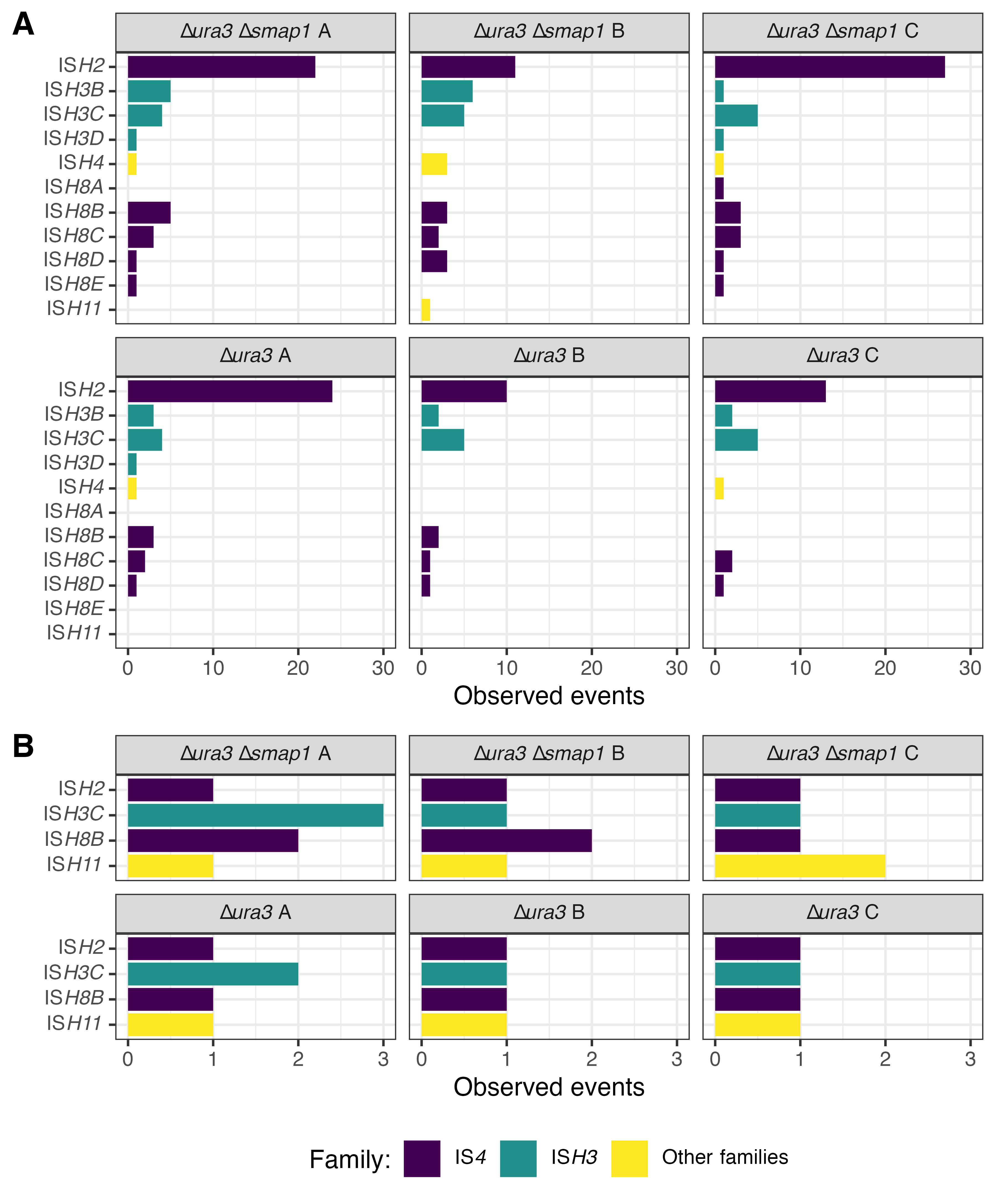

Supplement: FIG S5 [file msystems.00816-22-s0005.tif]

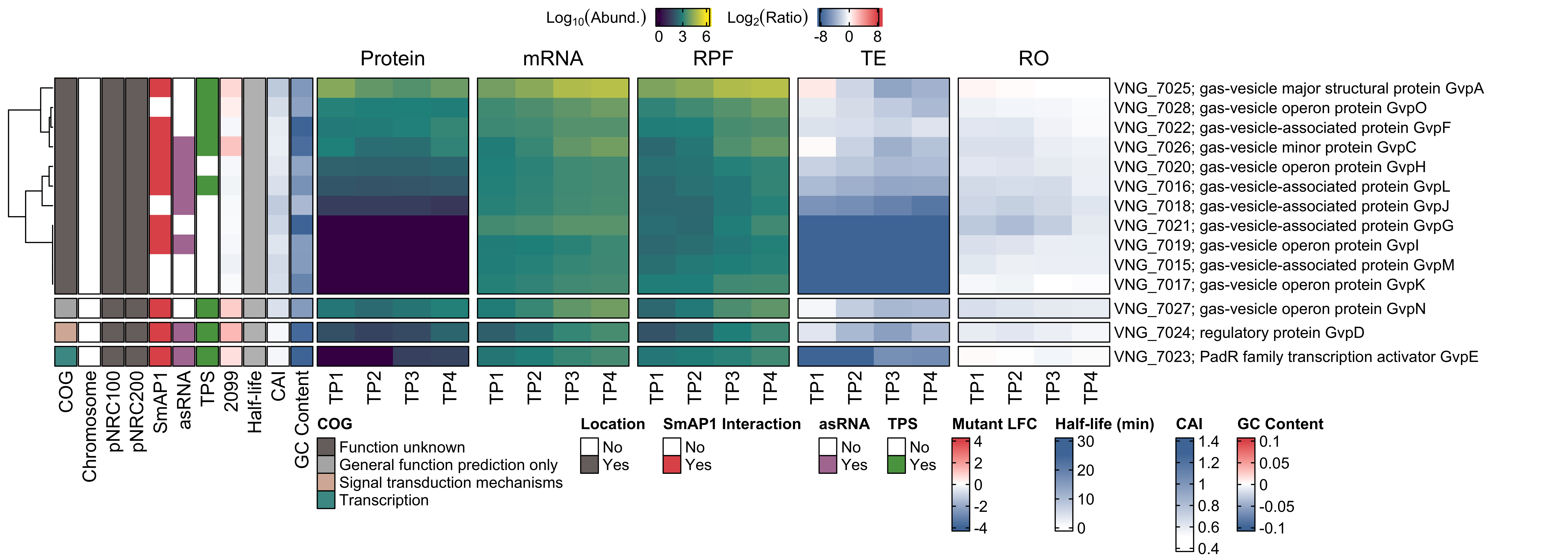

Supplement: FIG S6 [file msystems.00816-22-s0006.tiff]

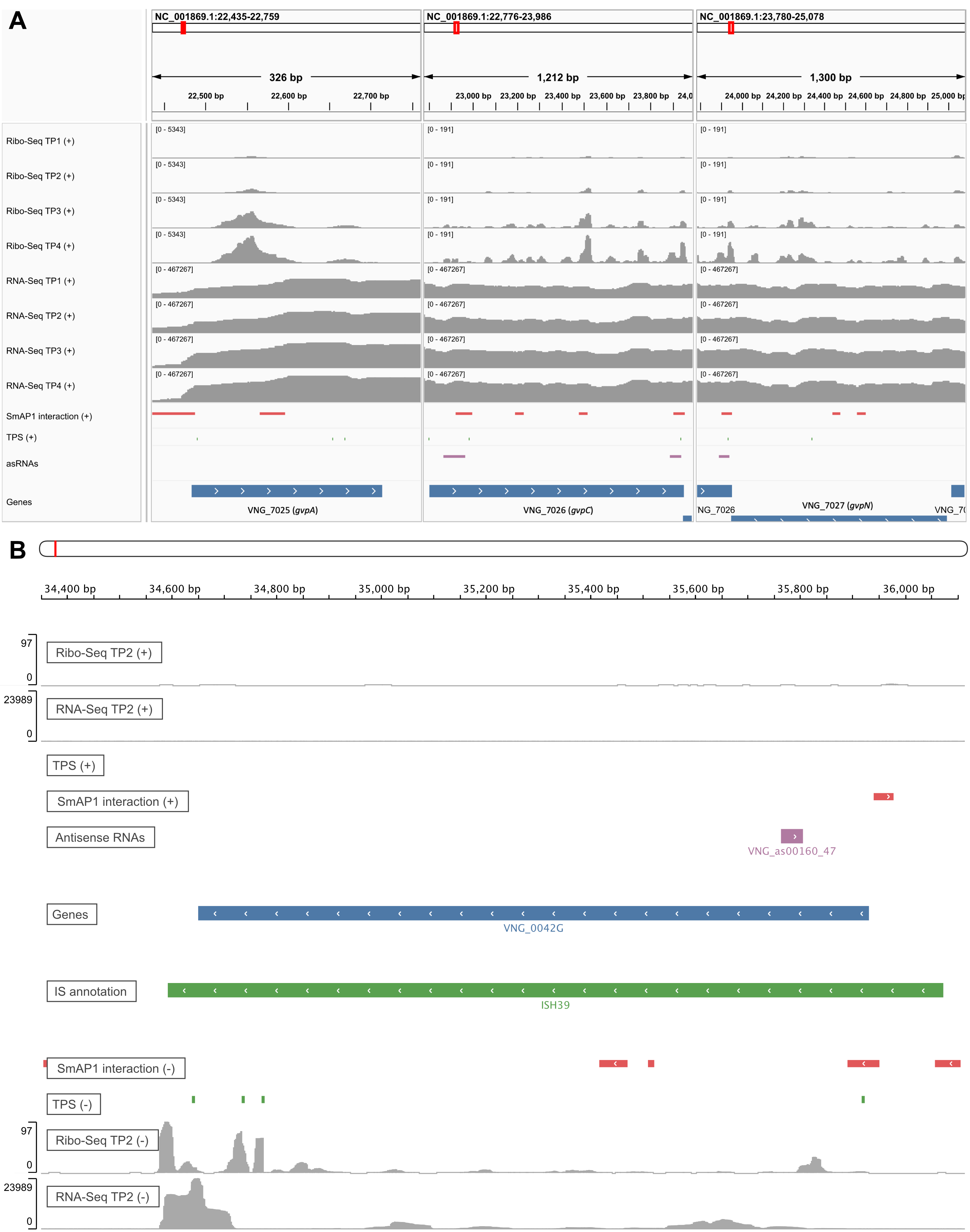

Supplement: FIG S7 [file msystems.00816-22-s0007.tif]

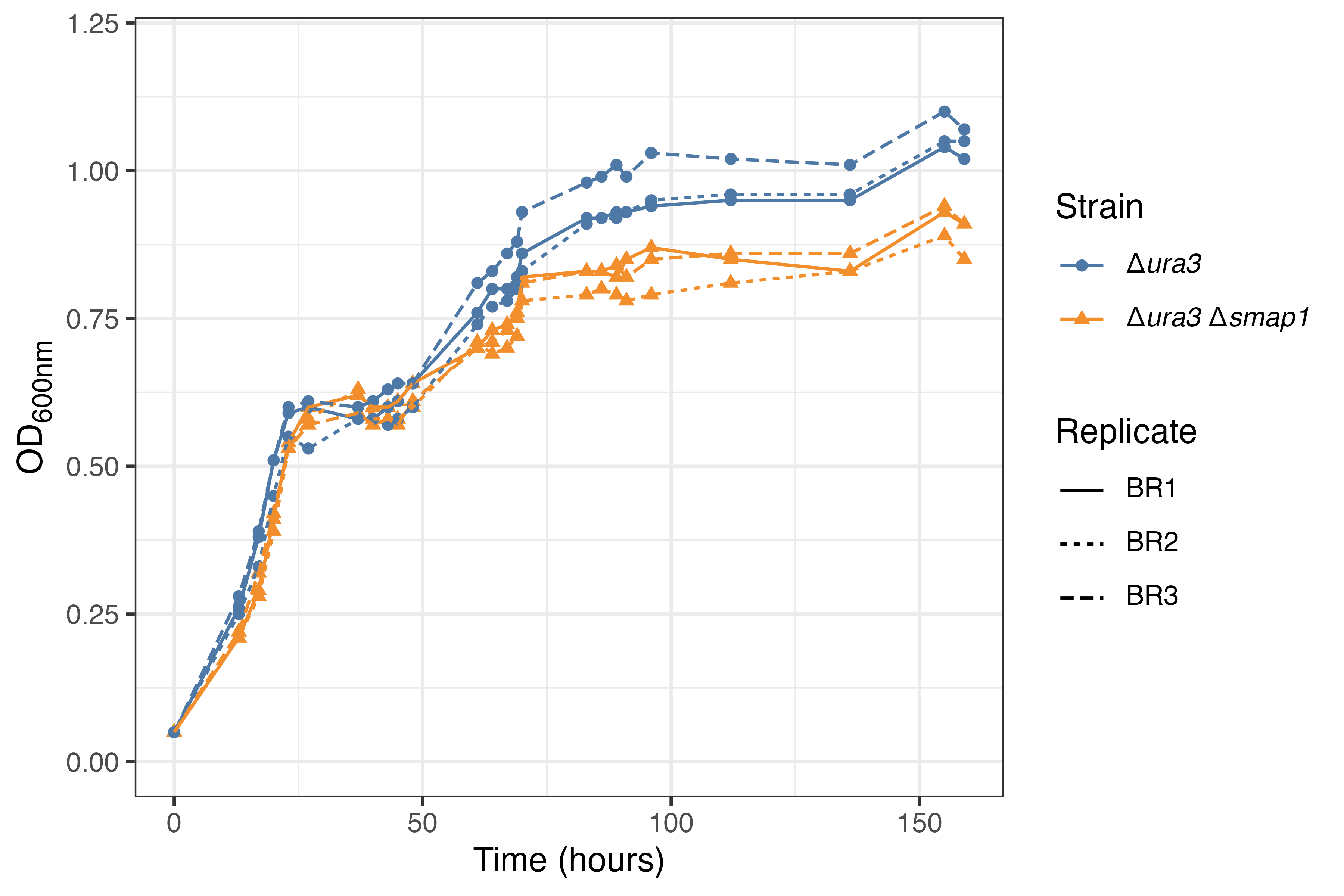

Supplement: FIG S8 [file msystems.00816-22-s0008.tif]
